# A comprehensive review of drying meat products and the associated effects and changes

**DOI:** 10.3389/fnut.2022.1057366

**Published:** 2022-11-28

**Authors:** Ahmed Mediani, Hamizah Shahirah Hamezah, Faidruz Azura Jam, Nursyah Fitri Mahadi, Sharon Xi Ying Chan, Emelda Rosseleena Rohani, Noor Hanini Che Lah, Ummi Kalthum Azlan, Nur Aisyah Khairul Annuar, Nur Aida Fatin Azman, Hamidun Bunawan, Murni Nazira Sarian, Nurkhalida Kamal, Faridah Abas

**Affiliations:** ^1^Institute of Systems Biology, Universiti Kebangsaan Malaysia (UKM), Bangi, Selangor, Malaysia; ^2^Manipal University College Malaysia (MUCM), Malacca, Malaysia; ^3^Faculty of Science, Universiti Teknologi Malaysia, Johor Bahru, Johor, Malaysia; ^4^Faculty of Information Science and Technology, Multimedia University, Malacca, Malaysia; ^5^Department of Food Science, Faculty of Food Science and Technology, Universiti Putra Malaysia, Serdang, Selangor, Malaysia

**Keywords:** dried meat, health benefits, sensory and functional attributes, safety, biochemical and nutritional compositions

## Abstract

Preserving fresh food, such as meat, is significant in the effort of combating global food scarcity. Meat drying is a common way of preserving meat with a rich history in many cultures around the globe. In modern days, dried meat has become a well enjoyed food product in the market because of its long shelf-life, taste and health benefits. This review aims to compile information on how the types of meat, ingredients and the used drying technologies influence the characteristics of dried meat in physicochemical, microbial, biochemical and safety features along with technological future prospects in the dried meat industry. The quality of dried meat can be influenced by a variety of factors, including its production conditions and the major biochemical changes that occur throughout the drying process, which are also discussed in this review. Additionally, the sensory attributes of dried meat are also reviewed, whereby the texture of meat and the preference of the market are emphasized. There are other aspects and concerning issues that are suggested for future studies. It is well-known that reducing the water content in meat helps in preventing microbial growth, which in turn prevents the presence of harmful substances in meat. However, drying the meat can change the characteristics of the meat itself, making consumers concerned on whether dried meat is safe to be consumed on a regular basis. It is important to consider the role of microbial enzymes and microbes in the preservation of their flavor when discussing dried meats and dried meat products. The sensory, microbiological, and safety elements of dried meat are also affected by these distinctive changes, which revolve around customer preferences and health concerns, particularly how drying is efficient in eliminating/reducing hazardous bacteria from the fish. Interestingly, some studies have concentrated on increasing the efficiency of dried meat production to produce a safer range of dried meat products with less effort and time. This review compiled important information from all available online research databases. This review may help the food sector in improving the efficiency and safety of meat drying, reducing food waste, while maintaining the quality and nutritional content of dried meat.

## Introduction

Fresh meat is a rich source of protein and it is very fast in spoilage. Thus, people from all over the world have come up with ways to preserve and enhance the flavors of meat by drying the meat. For instance, “Pastirma” in Turkey, “jerky” in North America, “carne-de-sol” in Brazil, “biltong” in South Africa, “kaddid” in North Africa, and “cecina” in Spain are only few of many types of dried meat products from all over the globe. Multiple meat drying methods are applied, including hot air drying, cold drying, sun drying, vacuum drying, ultrasonic drying, freeze-drying, microwave drying, heat pump drying, pulsed electric field drying, and refractance window drying (1). Although these techniques are effective in drying meat, the ones that require higher temperatures are claimed to degrade valuable nutrients in the meat while also diminishing the quality of the dried meat (1). Nevertheless, dried meat products come in diverse types, taking into consideration the types of meat, drying methods and presence of spices.

For instance, springbok, kudu, gemsbok, and ostrich are common dried meat products in South Africa, known locally as biltong (2). In addition, biltong had a rich history in terms of processing, in which South Africans used to dry hunted meat in the sun, occasionally adding spices and vinegar made from grapes of the French Huguenots to the drying meat (3). Traditionally, the meat would be dried for a couple of weeks before being exported, with the dried meat wrapped in cloth (1, 3). A study studied the fatty acid, iron and peptide contents in dried ostrich, beef and chicken meats and concluded that dried ostrich contained four times less fat compared to beef and two times less fat compared to chicken (2). In modern times, dried meat is used as a convenient addition of protein and flavor, especially observed in instant noodle formulations whereby dried chicken cubes are incorporated into packaged meals (4). The traditional air-drying technique was further investigated in which the traditional method of drying was considered less effective as it was energy and time-consuming. They mentioned that higher temperatures could lessen the time and energy required for the meat drying process. However, it was also stated that higher temperatures diminished the flavor of the meat and degraded its nutritional compounds while also hardening the final product (4). Ultimately, it was found that air-drying meat at lower temperatures could be done at lower temperatures and shorter times if the interaction between myofibrillar protein and water in the meat and in this case, lamb meat is considered.

In Inner Mongolia, Qinghai, dried beef, and yak are manufactured as popular snacks. Common dried meat snacks are produced in the current market by ancient air-drying technologies passed down thousands of years ago (5). The dried meat products varied widely with factors, such as the breed of the ruminant, presence of spices and processing methods used, which include the ancient technique of natural air-drying, drying and frying as well as drying and roasting (5). These factors were investigated in that study for their effect on the formation of heterocyclic aromatic amines (HAAs), a carcinogenic compound, in dried meat products. The results showed that beef that was dried by roasting contained higher HAA contents, while deep-fried beef had the lowest HAA content. At the same time, the study found that spices inhibited the formation of HAA in air-dried meat products and that dried meat from Chinese yellow cattle had higher levels of HAA compared to dried Aberdeen Angus cattle and Chinese yak meat (5). Table 1 summarizes the type of dried meat according to their drying methods.

**TABLE 1:** Summary of the type of dried meat according to their drying methods.

Product	Type of meat	Ingredients	Method of drying	Ref no.
Biltong	- Springbok - Kudu - Gemsbok - Ostrich	- Salt - Vinegar - Black pepper - Coriander - Brown sugar	Hot air/sun drying	(2, 3)
Dried chicken	Chicken	N/A	- Superheated steam drying - Heat pump drying - Hot air drying	(6)
Jerky	- Lamb - Beef - Yak	Salt	- Air drying - Hot air drying	(4, 5, 7)
Sucuk	- Beef - Water buffalo - Lamb	- Salt - Nitrate/nitrite - Glucose - Black and red pepper - Cumin - Allspice - Garlic	Air drying	(8)
Pastirma	- Beef - Water buffalo	- Salt - Sucrose/glucose - Garlic - Red pepper	Dry during	(8)
Kadid	- Beef - Lamb - Camel	Salt	Sun drying	(9, 10)
Cecina	Beef Foal	Salt	Dry curing	(11, 12)
Carne-de-sol	Beef	Salt	Sun drying	(1)

With the diversity of dried meat products from different cultures, it is important to study the characteristics of the products in terms of the health benefits, ingredients, physicochemical and biochemical changes, as well as the safety challenges involved. Furthermore, the microbial characteristics of dried meat products are noteworthy since the main purpose of drying meat is to preserve the meat by preventing the growth of harmful microbes. In that sense, advanced technologies aiming to enhance the efficiencies of meat drying are also to be discussed.

## Health benefits of dried meat

While dried meat has the probability of being harmful, in which this will be discussed on as safety challenges, numerous studies have reported various health benefits of dried meat (2, 5). Similar to the health benefits of fresh meat, dried meat maintains the sought-after protein from fresh meat. This is also what makes dried meat popular as it is easier to store and usually in ready-to-eat packages for the convenience of consumers. As previously reported, carnosine and anserine are found in various dried meat products at differing levels (2). Carnosine and anserine are dipeptides that are commonly found in fresh meat, especially red meats. The same dipeptides are found in dried meat products as well, preserving the health benefits of fresh meat (2). Carnosine is known to have potential in heart failure treatment as it is found to be related to cardiac cell contractions (13). Meanwhile, anserine is a derivative of carnosine that is transformed reversibly back to carnosine by multiple enzymes (13). Furthermore, dried ostrich meat had the highest anserine content while beef and chicken had higher carnosine contents instead, with beef having higher levels of carnosine. However, carnosine and anserine contents were lower in all types of spiced dried meat (2).

**FIGURE 1:**
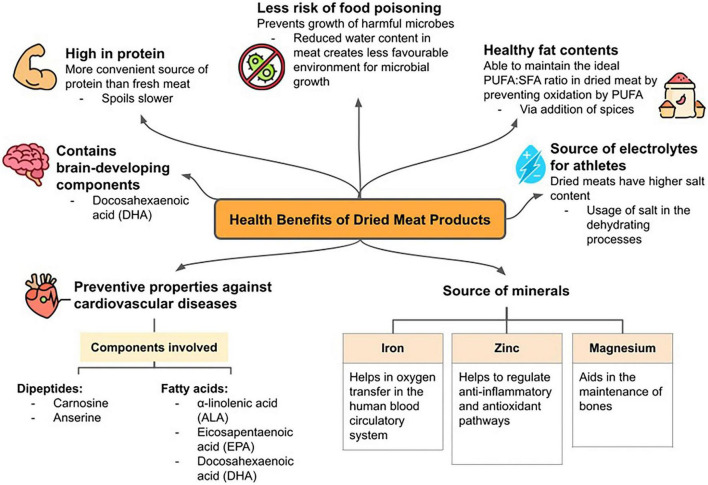
Health benefits of dried meat.

Other than that, fatty acids α-linolenic acid (ALA), eicosapentaenoic acid (EPA), and docosahexaenoic acid (DHA) were found in dried meat products (Figure 1), with dried ostrich meat having the highest concentration of all mentioned fatty acids compared to dried beef and chicken (2). The three fatty acids are known to have preventive properties against cardiovascular diseases (14). Furthermore, DHA was reported to aid in brain development as well (2, 15, 16). The dried ostrich and chicken meat had healthier polyunsaturated fatty acid (PUFA): saturated fatty acid (SFA) ratios compared to dried beef, whereby coronary heart diseases could be prevented with optimal PUFA:SFA ratios (2). However, the study has also suggested that the addition of spices could help in maintaining the ideal ratio since herbs and spices could reduce the oxidative process of overly high levels of PUFA in dried meat products with long shelf lives. Additionally, minerals are also abundant in dried meat products, especially iron, zinc, and magnesium. It was also reported that dried ostrich contains twice as much iron content compared to dried beef and 20 times more compared to dried chicken (2). However, it was found that higher temperatures reduce the levels of available iron in dried meats, and lower temperatures were recommended for the drying process. Since iron is compulsory for oxygen transfer in the human blood circulatory system, dried meat could be considered a valuable source of this mineral.

At the same time, zinc and magnesium were proved in another study to be of nutritional value in dried meat, with higher levels of zinc in dry fermented sausages and magnesium more abundant in other dried meat products (17). As micronutrients, zinc and magnesium serve significant purposes in human health. Zinc can regulate anti-inflammatory and antioxidant pathways and supplementary doses of zinc are also used to treat health issues like liver disease, cancer, heart disease (18). On the other hand, magnesium serves in the maintenance of bones by regulating calcium and phosphate homeostasis via the activation of the vitamin D (19). Dried pork was found to have higher zinc and magnesium contents compared to dried chicken (17). Nevertheless, the presence of zinc and magnesium in dried meat products is relieving for consumers with the health benefits that come with the micronutrients. All in all, dried meat products bring a variety of health benefits to consumers, with the level of beneficiaries differing among the type of meats used. This of course is also affected by factors like the drying process and the presence of additional spices.

## Dried meat and meat products

Dehydrated meat is referred to as whole muscle or crushed and formed meat products that have undergone dehydration to remove water content in the meat that will hinder the growth of microbial and biochemical reactions to prolong the shelf-life and improve the quality of the meats (20). Cured ham, biltong, beef jerky, and pastirma are examples of air-dried meat products that are most found these days (21). From then until now, foods, including meats have been dehydrated or dried by various drying techniques. The most conventional and broadly used technique of drying, natural sun drying, started to be replaced by mechanical drying (22). There are four generations of dryers, which first-generation dryers consist of cabinet and bed type dryers, including kiln, truck trays, tunnel, and rotary dryers (21). Second-generation dryers comprise spray and drum dryers, which are meant for dehydrated powders and flakes. Meanwhile, third-generation dryers, the use of freeze and osmotic dryers for plasma and biological products. The latest one, fourth-generation dehydration technology implies high vacuum, fluidization, and use of microwaves and radio frequency.

Meat products are foods that primarily contain meat and may also contain fat, water, salt, curing agents, spices, and other ingredients. Since the beginning of time, meat has been processed and turned into a range of products with the aim of preserving it for a long time, making the most of the entire carcass, improving taste and diversity, and improving convenience (23). There is another type of dried meat which is intermediate-moisture meats. This type of dried meat is a stable temperature product consisting of moisture content around the equilibrium moisture content of the meat mixture at room temperature and humidity (24). The intermediate-moisture meats have a desirable texture without being too dry or brittle, so that they may be ingested without being rehydrated. The majority of conventional intermediate meat products have developed as a result of the meat mixture’s natural drying process. During the processing of fresh foods with intermediate moisture levels, some water is removed, and the availability of the remaining water is decreased by the addition of appropriate solutes (25). In addition, intermediate-moisture meats do not require to be stored in refrigeration or in packaging that is strictly moisture-proof. In fact, the goods have been dried and treated to a stable state, so that they may be stored at room temperature without getting spoiled.

### Advanced drying technologies for drying meat and meat products

In conventional drying processes, thermal deterioration reduces the drying rate and rehydration ratio, resulting in loss of flavor, color, and nutrients, which include vitamins and amino acids (26). As a result, advanced technologies should be applied to produce higher quality drying and dried meat products. The porosity structure and bulk densities of dried meat products are all affected by the drying processes (27). Loss of water and application of heat can lead to the formation of pores and shrinkage which can affect the texture and taste of the meats (28). In a study, minced meat was dried using advanced drying techniques such as vacuum drying, ultrasound-assisted vacuum drying (USV), and freeze-drying (29). During the drying process, these approaches possess various benefits.

In vacuum-drying, the low temperature and absence of oxygen can preserve heat-sensitive and rapidly oxidizable foods. Therefore, discoloration and loss of flavor and some nutritional components can be avoided (30). However, the vacuum-drying does not entirely prevent the quality loss in dried meat (29). Thus, alternative methods, including ultrasound, microwave, and freeze, may minimize drying time and quality loss. On the other hand, the USV technology combines ultrasonic treatment and vacuum drying to reduce the drying time. The suction process, by breaking the cell tissues of the food, stimulates water transfer, thus increasing the drying rate. The mechanical waves created by ultrasonic treatment contribute to the movement of heat and water from within the meat to the surface. Additionally, the pressure and frequency of the ultrasonic waves significantly affect the transfer rate (30). This approach produces consistent drying and allows the process to be completed in a shorter time. To maintain the meat was dried safely, the air pressure is kept very low and the boiling point of water is less than 40°C, the minimum temperature of heat that begins to disrupt the cell tissue (31). Another mechanism used during ultrasonic drying is cavitation, which accelerates water transport from the removal of tightly bound water. Ultrasonic modifications in meat drying may be characterized as having a higher drying rate, preventing loss of food quality, decreasing energy consumption and process cost, and altering the meat composition (32).

Furthermore, freeze-drying is one of the ideal drying methods for the biological matter that are susceptible to heat and oxidation. The freeze-drying principle was based on the three-state transition of water. The triple point temperature of the water is 0.0098°C, and the pressure is 4.579 mmHg (33). In this process, water in food was frozen at a low temperature and immediately sublimated from solid to gas in a vacuum. Thus, freeze-dried foods retain their nutritious value as it does not promote protein denaturation or vitamin loss (34). It preserves the dried meat’s original color, flavor, aroma, and appearance to the maximum degree possible while protecting its composition, making it particularly suitable for drying meat products (35). The product of freeze-dried methods has low moisture content and can be reconstituted with a high rehydration speed (33). Moreover, dried meat products with a porous structure and good rehydration capabilities were also attainable using freeze-drying approaches (36).

In choosing the proper drying process, optimizing the drying temperature is an essential element influencing the quality of dried meat products. Drying periods can be reduced by rapid heat and moisture transfer at high temperatures. On the other hand, high temperature may cause the breakdown of some heat-sensitive compounds, such as proteins and vitamins, as well as a decline in color quality and rehydration capacity and an increase in shrinkage rates (37, 38). Although the drying period may be prolonged, low-temperature drying methods, such as freeze drying, are recommended for drying meat products as it prevents microbiological and biochemical degradation (39). A drying mechanism with a temperature that does not affect the meat’s physicochemical, nutritional, or sensory quality with sufficient drying time should be chosen.

### Drying condition and its role in meats quality

Drying is one of the most crucial food preservation techniques, which involves reducing the moisture levels of products to the necessary moisture content in order to avoid spoiling and retain quality (40). It is a process that simultaneously transfers heat and moisture, which causes changes in the product that is being dried (41). Because it is effective and affordable, drying is one of the oldest and most popular preservation techniques (29). Meat drying is the removal of water content from the meat surface in order to obtain an ideal condition for dried meat product preservation. Drying conditions should be thoroughly considered as they will affect the quality of the meats after going through some processes which may alter the quality or condition of the meats that people desire to preserve. Temperature, drying period, relative humidity, and water content are crucial parameters in the drying condition that will affect the quality of the dried meats (21). A lot of nutritional loss, which includes degradation of vitamins and amino acids, as well as flavor and color loss occur due to thermal degradation that decreases the drying rate and rehydration ratio when using traditional thermal drying techniques (26). Therefore, the application of advanced and newest technologies should be implemented in order to acquire a better quality of dried meat products (29).

The advantage of applying high temperature is that less drying period will be required because of more rapid heat and moisture transfer, but the high temperature may contribute to the deterioration of compounds that are heat-sensitive, such as protein and vitamins (29). On the other hand, low temperatures are recommended in drying processes as it will lessen biochemical and microbial decomposition (39). The application of low temperature in the absence of oxygen by using the vacuum drying technique can preserve heat-sensitive and easily oxidized foods (29). However, it is time-consuming as the drying duration will increase. Therefore, it is best to choose a drying temperature and drying duration that do not compromise a product’s physicochemical, nutritional, or sensory qualities. Based on a previous study, it was observed that drying temperatures and periods had affected the moisture content and water activity (42). Moisture content and water activity of ground dried meat declined as the drying period, and temperature increased. Controlling the moisture content and water activity of dried meat is important to avoid a favorable environment for microbial growth in the meat. Therefore, extensive exposure to a high humidity environment should be avoided to restrict the contamination of microorganisms.

Dry-cured ham meat products were found to have high quality when placed in curing chambers under mild conditions, at temperatures of 14–16°C for more than 8 months (43). After the meats had lost the predicted loss of moisture, a layer of lard was smeared on them as a way to prevent excessive dehydration. Meanwhile, meats of poorer quality were exposed to more intense drying conditions for shorter periods, where the temperatures rose to 25–28°C for a few months (43). The long processes with mild drying conditions enable a significantly higher enzyme activity, which in turn increases the production of free amino acids and free fatty acids (Table 2). Free amino acids directly result in giving taste to the meats and indirectly contribute to specific aroma compounds, whereas free unsaturated fatty acids are oxidized furtherly to produce aroma volatile compounds (44). Apart from temperature and drying period, relative humidity and moisture contents are also parameters in drying conditions that should be looked into, which will influence the quality of the dried meats. Microbial growth that can spoil the meat products can be minimized by reducing the water content of the meat (45). The most ideal humidity for drying conditions can be determined by testing several different humidity on the meat products. For instance, the three different relative humidity of drying air (52 ± 3, 78 ± 3, and 85 ± 3%), it was found that rapid dehydration at 52 ± 3% relative humidity (RH) can reduce the water content at the surface of the meat (46).

**TABLE 2:** Different drying techniques with their respective drying conditions.

Type of meat	Drying technique	Drying conditions	References
Hams	Convective drying	12 and 14°C for 1 month, at 16–18°C for 2 months and at 18–20°C for 5 months. The relative humidity ranged from 60 to 70%. Air velocity at 0.1 and 0.5 m/s	(46)
Chicken, beef, and chevon strips	Sun-drying Oven-drying	33°C for 120 h 60°C for 72 h	(40)
Kilishi (intermediate meat) prepared from beef and pork	Sun-drying	29–31°C for 7 h (1–2 days)	(47)
Beef and chicken meats	Ultrasonic vacuum drying Vacuum drying Oven drying	Temperature at 55, 65, and 75°C	(37)
Pork ham	Oven-drying	40, 50, 60°C for 5, 6, 7, 8 h	(42)
Broiler chicken breast meat	Freeze drying	Slow freezing 20.5 h of primary drying (12 h at 0°C and 8.5 h at 10°C) at 30 Pa.	(48)
Beaf meat	Convective drying Vacuum drying Microwave vacuum drying	Temperature 40°C, relative humidity 30% Vacuum pump flow rate 350 m^3^h^–1^, temperature 40°C Vacuum pump flow rate 350 m^3^h^–1^ Microwave power 300 W Temperature 40°C Pressure 5.3 kPa	(49)
Pork tenderloin	Microwave vacuum drying and puffing	Temperature at 70°C and air speed at velocity of 1.1 m/s.	(50)
Turkey breast meat	Air drying Microwave Freeze drying	Temperature 60, 75, 90°C, air velocity 1.8 m/s Microwave power 180, 360, 540 W Frozen at −18°C for 24 h. Vacuum pressure at 0.1, 0.15, 0.2 mbar	(51)
Beaf meat	Microwave-assisted hot air drying	Fixed air speed (1.0 m/s) Air temperature (60 and 70°C) Microwave power (0, 90, and 180 W)	(52)

## Ingredients used in dried meat and meat products

Other than the meat itself as the main ingredient for dehydrated meat products, several ingredients such as salt, spices and additives are commonly added. Generally, these added ingredients aid in the meat processing, increase the meat’s sensory qualities and increase the products shelf life (Table 3). Although most of the added ingredients are in solid forms (powder or coarse grind), it can also be in a liquid form, such as essential oils. Other than food safety and added sensory qualities, the added ingredients can also contribute to the health benefits of the final meat products.

**TABLE 3:** Non-meat ingredients added during the dried meat process.

Ingredients	Function/Role	Form	Range	Safety/Toxicity	Products	References
Salt (NaCl)	Osmotic dehydration of minced meat Lowered the water activity, a_w_ via increased salt uptake by the meat	Crystals	20–25%, 5% with maltodextrin 40%	Upper limit is 26% NaCl, where higher concentration caused no significance water loss	Dehydrated minced beef meat, dried pork loins	(53–55)
Oregano and thyme essential oil	Maintained a stable microbial activity in the meats during the storage period Contributed to some sensorial properties Extending the shelf life of meat	Essential oil	0.15–3.0 mg/kg per day	Establishment of three classes of toxic constituents, where the first class has the higher upper limit and vice versa for lower limit of the daily intake	Dehydrated pork meat and products	(20, 56, 57)
Black pepper and red pepper	As seasoning to the meat	Powder or peppercorns	83–333 mg daily intake for black pepper	No significant effect as per studied	Dried beef chips	(52, 58)
Potassium sorbate	Act as preservatives by inactivating *Salmonella* spp. and *Listeria monocytogenes*	Powder and in washing	<25 mg/kg	>25 mg/kg may cause genotoxic and cytotoxic effect	Beef jerky, sheep meat, poultry	(59)
Galangal, coriander, garlic, thyme	Lower the reactivity of nitrite and antioxidant activity	Stems and leaves	Not mentioned	Toxicity mostly caused by the heavy metals and pesticides residues in the plant	Dendeng, beef	(60)
Nitrite (sodium/potassium nitrite)	Give red color to the dry cured meat Act as an anti-botulinal agent	Crystal	<150 μg/ml for meat products	Consumption higher than the upper limit may lead to blue baby syndrome	Dry cured ham, dry cured sausage	(61–63)

### Salt

The most commonly added ingredient in dried meat products is salt or sodium chloride, NaCl (52, 53). Salt is essential not only as a seasoning to the meat, but also for its dehydrating effect on the meat for preservation. There are various instances where salt is used for the purpose of dehydrating meat. In salt, together with water is a binary osmotic treatment on meat, causing osmotic dehydration of the meat. This treatment can lead to osmotic dehydration, where water is removed from the meat in a non-thermal manner. High concentrations of NaCl in the osmotic or brine solution cause the salt uptake of the meat to be increased and decreased in the water activity (a_w_) (53, 54). Thus, the addition of salt in the processing of meat can decrease the growth of salt-sensitive microorganisms, due to the lower a_w_. This is demonstrated by Dimakopoulou-Papazoglou and Katsanidis (53) by using NaCl treatment together with maltodextrin, to the dehydrated beef (53). The treatment with maltodextrin instead of only using NaCl has been shown to preserve the red color of the meat. Consequently, low a_w_ and lower microbial load cause higher storage stability of the meat product, which contributes to extended shelf life.

### Spices and herbs

Spice is defined differently in different regions of the world. Based on Peter and Shylaja (64), spice is the various parts of the plant, except leaves, that have been dried (64). These plant parts give flavors to the food it is added into and are sometimes pungent. On the other hand, herbs can be defined as the dried leaves of fragrant plants that are used to flavor and odorize meals. The plant stems and leaf stalks are frequently traded separately from the leaves (64). Nonetheless, there are various types of spices and herbs that can be added during the processing of dried meat, where it varies on the types of meat products. Tropical plants that have the pungency or flavor of aromatic substances are known as spices. To improve the flavor of food, spices can typically be added as whole spices, ground spices, or spice extracts (65). Spices and condiments such as black pepper, red pepper, anises, and cardamom are added to increase the sensory qualities of the meat (52). These spices have antioxidant, antifungal, and antimicrobial effects, which are beneficial for lengthening the shelf-life of dried meats (65).

Herbs in the form of edible oregano and thyme essential oil are added for their benefits. Oregano essential oil, specifically, maintains a stable microbial activity in the meats during the storage period. It also contributes to some sensory properties of meat products. Interestingly, the oregano essential oil together with the starch edible coating is used in various studies of the meat-drying process in order to increase meat quality alongside packaging methods (20). Garlic and thyme are also herbs that can be used in marinating meat products before drying. Both of these herbs have been shown effective on mesophilic aerobic bacteria and lactic acid bacteria (66). This shows that herbs and spices can be used as natural preservatives for meat products.

Moreover, there is good evidence that shows garlic can potentially lower total cholesterol, low-density lipoprotein, high-density lipoprotein, and triglycerides (67). Other spices and herbs also have their advantages to health. As an example, thyme essential oil is highly potential to be used for osteoporosis prevention, as it is shown to inhibit bone resorption and bone inflammation in rats (68). On the other hand, black pepper, *Piper nigrum* Linn, has been studied and shown to have potential anti-inflammatory effects (69). Therefore, spices, herbs, and condiments are important in incorporation into dried or dehydrated meat to add flavor, antimicrobial, antifungal, and antioxidant properties, as well as vital health benefits.

### Additives

Additives are added to the dried meat and meat products during the processing, usually to achieve the desired color or as preservatives. One of the preservatives commonly used is potassium sorbate, which is added or used in washing sheep meat, beef jerky, and poultry. The main goal of potassium sorbate is as a preservative agent by reducing or inhibiting the activity of microorganisms, which in turn will extend the shelf life of the meat products. The antimicrobial effects have been shown for *Salmonella* spp. and *Listeria monocytogenes* (59, 70, 71). The addition of potassium sorbate, together with other factors such as pH, a_w_, and temperature, was shown to be successful in the thermal inactivation of *Salmonella* spp. (59). For *L. monocytogenes*, washing the meat with potassium sorbate caused reduced microbial activity, which extended the product shelf life for 2 days longer (70). Therefore, potassium sorbate is a preservative, mainly added to the meat for its preservation properties.

Other than that, nitrite is also a common preservative used in meat products. Similar to potassium sorbate, nitrite plays a role as a bacteriostatic, bacteriocidal, and antibotulinal agent for *Clostridium botulinum*. As an example, in cured meat products, it is added as a strong inhibitor to anaerobic microbes, especially *C. botulinum* (72). Moreover, nitrite also acts as a coloring agent when added to meat products, as it is a strong heme oxidant. Generally, nitrite acts as an oxidizing agent to the heme in cured meat products to achieve the red color of typical meat products (62, 72). The usage of nitrite as a curing agent has been shown to contribute to a better aroma in dry fermented sausage products. This is determined by the detection of odor-active compounds such as hexanal, heptanal, and 1-octen-3-ol, which were proposed as the compounds contributing to the aroma of dry fermented sausages (73). Thus, the addition of nitrite and potassium is crucial as a preservative and coloring agent to meat products, although alternatives using spices and herbs can also be implemented using garlic, thyme, and spices (66).

## Physicochemical characteristics

**FIGURE 2:**
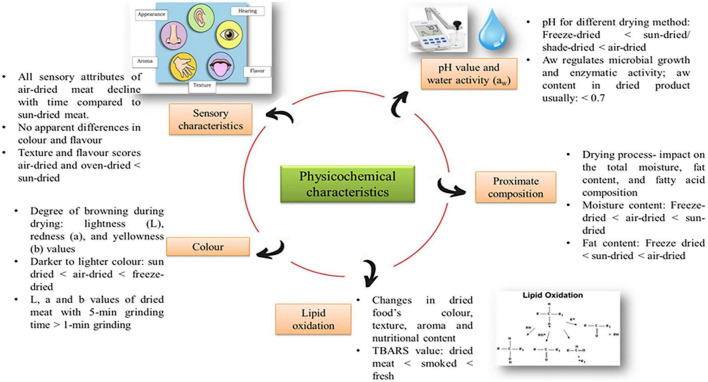
The summary figure of physicochemical characteristics of dried meat and meat products.

Physicochemical characteristics are referred to the intrinsic physical and chemical aspects of dried meat products. The physicochemical characteristics of dried meat include pH, water activity, lipid oxidation, proximate composition, color, and sensory characteristics (Figure 2). Dried meat quality is determined by a variety of physical features, including the amount of intramuscular fat and the age of the animal used (21). The surface texture of the dried meat may play a crucial function in consumer meat quality assessment. The dried meat’s chemical composition is also an essential aspect in determining its quality. The content of the meat varies according to the breed, species, and age of the animal (1). The distinctive quality of dried meat is mainly due to the chemical composition and physical structure of the meat (74). The physicochemical properties are essential indicators used in assessing the various drying techniques of dried meat.

### pH

pH is one of the essential characteristics of meat quality since it directly impacts the meat’s functional attributes, eating and storage quality (21). The pH of dried meat products is significantly affected by drying procedures. The pH of commercial dried meat samples ranged between pH 5.4–5.8 (75). Low pH values are important to avoid the denaturation of protein in the meat. The pH of dried and fresh biltong, and salted-dried South African meat had no significant difference. The pH value for dried biltong was 5.35 and fresh biltong was 5.58, respectively (74). The freeze-drying method was the most suitable method for producing dried meat with low pH. A study on different processes of dried meat revealed that among all the drying techniques, the freeze-drying method produced the lowest pH value at 5.89. In contrast, the air-drying method had the highest pH value at 6.08. The variation in pH values was caused by the loss of the free acidic group as the result of different drying techniques (21). In addition, a study on different drying methods for beef jerky discovered that hot air-dried jerky had a higher pH value than sun-dried and shade-dried. The air-dried method obtained a pH value of 6.25, while both the sun-dried and shade-dried processes had a pH value of 6.03. The elevated pH values appear to be caused by the denaturation of beef protein due to the drying processes (75).

### Water activity

One of the critical elements regulating microbial growth and enzymatic activity in dried food is water availability and location. The water activity coefficient, a measure of the thermodynamics chemical potential of water in the system, expresses the condition of water in the food (29). The water activity (a_w_) is defined as the ratio of the vapor pressure of water in food (p) to the vapor pressure of pure water (p0) at the same temperature (21). In dried products, the a_w_ contents are often less than 0.7 as it is responsible for microbiological stability to prevent microbial growth (76). In the study of beef jerky, all drying processes of meat, such as air-dried and sun-dried, achieved low a_w_ with values of less than 0.75, in which signifies low water contents (75). The water content and water activity are not directly related; however, as the water content decreases, the water activity also decreases. A dried biltong study showed low a_w_ ranging from 0.65 to 0.68 (74). The use of sodium chloride (NaCl) in the meat facilitates the drying process and lowers the a_w_ level of the meat (74). The salting process on the meat had a significant influence on the a_w_, which reduced from 0.98 ± 0.004 for 0% NaCl to 0.92 ± 0.004 for 10% NaCl. The presence of NaCl in high amounts will lower the meat’s free water content, resulting in a lower a_w_ value. Moreover, the presence of active water may be more significant for food stability than the overall amount of water in the food (76). Furthermore, lipid oxidation is most significant at very high and very low a_w_ as there is efficient movement of pro-oxidants, in which oxidation increases in the latter (76). Water suppresses lipid oxidation in the early stage but stimulates the subsequent reaction of lipid breakdown products with protein as a_w_ increases (21).

### Lipid oxidation

Lipid oxidation causes significant changes during food storage and manufacturing, which leads to rancidity. Lipid oxidation can alter the dried food’s color, texture, aroma and nutritional content (21). Lipid oxidation often occurs during the cooking and storage stage. The level of ferrous ions in dried meat is considerably elevated after drying due to an increase in non-heme iron and the breakdown of heme pigments facilitating auto-oxidation and resulting in rancidity (21). The oxidation of lipids may be related to protein denaturation, antioxidant degradation, and enzyme activity (77). According to several studies, the thiobarbituric acid (TBA) assay is the most widely used technique for assessing the oxidative degradation of lipids in the muscle foods (21). The TBA value rises with an increase in fat content in the food. Pre-cooking the meat also raises the TBA value of dried meat products (77). The thiobarbituric acid reactive substances (TBARS) value is the most often used indicator for determining the degree of lipid oxidation in meat products (75). Dried meat products have a higher TBARS value (8.33 μg/g) than fresh (0.11 μg/g) and smoked (0.21 μg/g) meat products (21). The TBARS value in dried meat products is higher due to mincing, mixing, and drying processes, which result in an extensive breakdown of cellular structures that allow the fusion of meat and pro-oxidants (21). The TBARS value was used to measure lipid oxidation, ranging from 0.60 to 0.8 mg/kg. The sun-dried meat at 0.77 mg/kg produced a lower TBARS content than air-dried meat at 0.68 mg/kg, suggesting that the sun-dried meat is more susceptible to lipid oxidation (75). In contrast, the TBA development in dried meat products can be delayed in low-fat content meat and under good storage conditions (21). Moreover, the peroxide value of dehydrated meat products is significantly increased by freeze-drying methods. The freeze-dried meat produced the highest value at 83.3 mEq/kg, whereas air-dried meat produced the lowest value at 20.8 mEq/kg (1).

### Proximate composition

Proximate composition is linked to the content of moisture, protein, ash, and carbohydrate in dried food products. It is essential to note the amount of proximate composition to ensure that the drying process produces the best quality of dried meat (78). The proximate analysis will provide a comparison between drying techniques of dried meat based on specific nutrients. The drying process has an impact on the total moisture, and fat content of dried meat products (2). Generally, the drying process will cause a reduction in the moisture content, ranging from 4 to 5%, and the total fat content around 3–4% (21). The addition of condiments such as salt to dried meat products can elevate the ash level while lower the moisture level of the product (78). In 0% of salt, the moisture content was 72.98 ± 0.48% and the ash content was 1.17 ± 0.04%. However, the total moisture and ash content in 10% salt was 65.90 ± 1.11% and 7.80 ± 0.93%. The observed composition changes correlate to a decrease in water content induced by the action of the salt under osmotic pressure between the meat’s muscle cells during the drying processes (78).

Moreover, the differences in drying techniques have different effects on the dried meat’s proximate composition, such as total moisture and fat content (75). A study conducted on lamb strips processed by a freeze-drying method found that the amount of moisture was 4.41% and fat content was 3.54% (21). In the process of drying the meat, the freeze-drying method is a more effective way to reduce the moisture and fat content of the lamb compared to the air-dried method. It is revealed that freeze-dried lamb (4.41%) has less moisture compared to air-dried lamb (5.03%). For the total fat content, the air-drying method had a lower total fat content of 4.39% compared to the sun-drying at 15.06% and oven-dried at 15.97% (21). Mgbemere et al. (79) conducted a study on the quality of oven-dried beef slices by measuring their proximate composition (79). Meanwhile, Chukwu and Imodiboh (80) observed the changes in the proximate composition of beef slices using the process of sun-drying (80). It was discovered that the amount of total moisture in sun-dried method (5.99%) was lower than in oven-dried (11.6%). However, the values of ash, fat, protein, and carbohydrates were higher in sun-dried process than the oven-dried (79, 80). The result reflected that the sun-dried has a higher loss of moisture while effectively maintaining the amount of other nutrient composition as compared to oven-dried. The comparisons of proximate composition between various drying processes for dried beef and dried lamb products are shown in Table 4.

**TABLE 4:** Comparison of proximate composition of different drying techniques for dried meat products.

Parameters (%)	Freeze-dried	Sun-dried	Oven-dried	Air- dried

Dried lamb				
Moisture	4.41	n.a.	n. a.	5.03
Ash	11.67	1.55	1.39	7.46
Fat	3.54	15.06	15.97	4.39
Protein	n. a.	82.34	80.85	n. a.
Carbohydrates	n. a.	1.06	2.30	n. a.

**Dried beef**				

Moisture	1.15	5.99	11.6	n.a.
Ash	3.90	5.71	5.2	n.a.
Fat	23.90	24.28	11.4	18.2
Protein	62.82	61.95	49.8	59.3
Carbohydrates	n.a.	n.a.	18.9	n.a.

n.a., not available. The information was adapted from Zdanowska-Sasiadek et al. (2), Mishra et al. (21), Mgbemere et al. (79), Chukwu and Imodiboh (80) and Lee and Kim (2, 21, 79–81).

### Color

**FIGURE 3:**
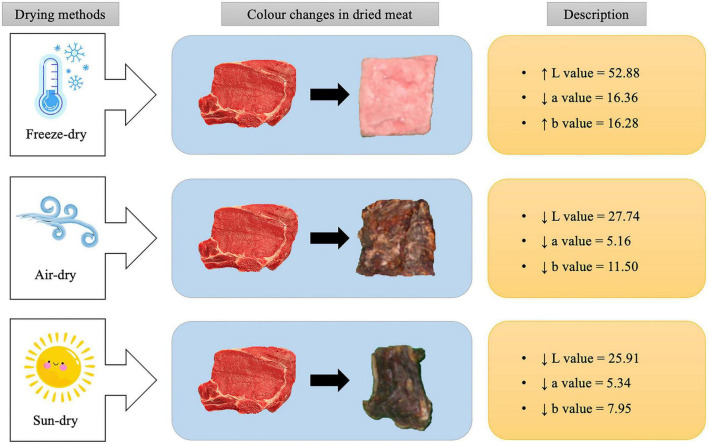
The color changes in dried meat under different drying methods.

Color is a vital attribute in food evaluation since consumers may immediately appraise it. The drying process has a significant influence on the lightness (L), redness (a), and yellowness (b) values, which are a source of variation in light scattering from the surface of the meat that represents the degree of browning during drying (82). In dried meat, the L value was in the range of 20–50, depending on the different drying methods. Meanwhile, the a value was between 6 and 10, and the b value was around 10–20. In dried meat products, a larger meat percentage increased the L, a, and b values (83). Furthermore, different drying techniques have an impact on the color of the products as well. Freeze-dried meat products exhibit a whiter color compared to the sun, air, or vacuum-dried meat products, which is primarily due to uniform light reflection from the surface of large pores (83). Additionally, the L, a and b values in fresh meat were found to be 48.46, 21.33, and 14.13, respectively (1). The freeze-dried meat produced higher L, a, and b values than other drying techniques, indicating that the freeze-drying had less effect on the protein structures of meat samples. As compared to freeze-drying, sun-drying methods give a darker brown color and rise in redness, indicating browning effects (21). The a value of air-dried meat (5.16) was lower than sun-dried meat (5.34). Nevertheless, the value of L and b for air-dried meat were 27.74 and 11.50, respectively, which was generally higher than sun-dried meat with an a value of 25.91 and a b value of 7.95 (1). The color changes in dried meat on three different drying methods are shown in Figure 3.

Furthermore, grinding is important in the food industry as it improves the surface area, giving a large surface area for chemical and biological activities (84). Dried meat prepared with a 5-min grinding time had higher L*, a, and b values than meat produced with a 1-min grinding time. The dried meat with a 5-min grinding time had an L value of 53.15 ± 2.27, a value of 5.99 ± 0.44, and b value of 10.62 ± 0.98. Meanwhile, the 1-min grinding time of dried meat had an L value of 48.50 ± 0.59, a value of 5.41 ± 0.17, and a b value of 8.35 ± 0.29 (83). The longer the grinding time, the bigger the surface area of the exposed meat to a high-temperature condition during the drying process. It significantly increases the browning of sugar amine due to the interaction of amine groups with the available reducing sugars in the meat connective tissues (83). In addition, chicken-based dried meat products have lower redness, yellowness, and light values than chevon, pork, and mutton-based dried meat products (21).

### Sensory characteristics

Sensory qualities were assessed for tenderness, color, taste, juiciness, and overall attractiveness of the meat (85). In the sensory evaluation and consumer acceptability, a score below 5 was described as less preferable, while a score above 5 was preferable among the consumers (1). The lower the score in sensory evaluation, the less preferable the dried meat method is. All sensory attributes of air-dried meat products decline with increasing storage time compared to sun-dried meat products. Lim et al. (75) conducted a study on beef jerky using the air-drying technique at a temperature of 80°C for 4 h, while sun-dried at 25–28°C for 3.5 h (75). It concluded that air-dried beef jerky scored 3.66 in the consumer’s overall acceptability while sun-dried meat scored 4.53, resulting in the sun-dried method being the preferable method in the drying process. The air-drying techniques can be attributed to excessive drying and moisture loss on the meat surface caused by high temperature during the air-drying process (75). However, the sun-dried beef jerky exhibited greater tenderness and juiciness compared to the air-dried beef jerky (75). On the other hand, there were no apparent differences in color and flavor between other drying processes. When compared to other techniques, such as air-drying and oven-drying processes, the texture and flavor scores of sun-dried meat products are comparatively higher (21). In air-dried meat, the high temperature from hot air leads to more hardness and chewiness, as well as the formation of off-flavors along the edges and corners of the meat.

## Microbiological characteristics of dried meat

**FIGURE 4:**
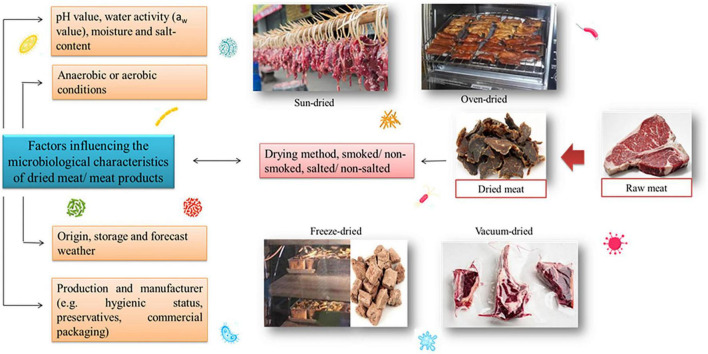
The multifactorial influences on microbiological characteristics of dried meat and meat products.

Microorganisms in dried meat and meat products are significant in determining the quality of the products and processes. Many attributable factors affect the microbial activities in dried meat (Figure 4), like the a_w_ values, which indicate the relative moisture balance or available water ratio that provides conditions for microorganisms’ growth. On the other hand, when rigid aerobic microorganism like pseudomonas is unable to grow under anaerobic conditions, lactic acid bacteria (LAB) will usually grow predominantly as the spoilage flora up to a maximum of 108/cm^2^ without yet spoiling the meat (86). However, the spoilage may be influenced by pH changes (>5.8) or oxygen contamination. As such, the microbiological characteristics of dried meat are usually described based on the standard total plate count (TPC) (<10^5^ cfu/g) for ready-to-eat food following the food safety guideline, lactic acid bacteria (LAB) and yeast and mold counts. These observations are highly associated with the water and salt content as well as the pH value of the dried meat. Many studies have been performed to evaluate the microbiological characteristics of the dried meat prepared in various preparation conditions, which includes microorganism like *Staphylococcus aureus*, *Salmonella* spp., *Bacillus cereus* and many others (74, 87).

Table 5 summaries the microbiological characterization of dried meat and meat products from various processing conditions, storage, packaging, and localities. The microbiological properties of meat products have been widely studied to ensure the development of safe food products for consumption. Biltong is a meat product processed through the dry-curing method, a traditional preservation method of meat product that removes moisture and decelerates a_w_, which consequently inhibits the growth of spoilage organism (88, 89). A study has evaluated the microbiological characteristics of various meat products from South Africa like the beef, kudu and springbok biltong samples (74). The investigated biltong samples were categorized into “dry biltong” (moisture content: 21.5–25.3 g/100 g; a_w_: 0.65–0.68) and “moist biltong” (moisture content: 35.1–42.8 g/100 g; a_w_: 0.85–0.89). An a_w_ value of 0.68 is the critical a_w_ for beef biltong to be microbiologically stable, thus higher than that is categorized as moist group, which allows microorganism growth for pathogens like *Listeria monocytogenes*, *S. aureus*, and *Staphylococcus pasteuri.* While there is no significant difference between both groups for pH value (5.00–6.26), distinct low TPC, high levels of LAB and D-lactic acid were observed in the “dry biltong” group suggesting that it adheres to the standard hygienic quality of meat products. Another compelling finding is the seeming co-existence of yeast and the micrococci counts that might suggest a synergistic potential of the co-existence, where one could be an inhibitory factor to another.

**TABLE 5:** The table summary of microbiological characterization of dried meat and meat products from various processing conditions, storage, packaging, and localities.

Dried meat/Meat products	Tested microorganisms	Conditions	Finding	References
Beef, kudu, and springbok Biltong, dried, cured meat	*L. monocytogenes*, *S. aureus*, and *S. pasteuri.*	- Dry biltong’ (moisture content: 21.5–25.3 g/100 g; a_w_: 0.65–0.68) - “Moist biltong” (moisture content: 35.1–42.8 g/100 g; a_w_: 0.85–0.89)	Distinct low TPC, high level of LAB and D-lactic acid was observed in “dry biltong” as compared to “moist biltong” group. Only *S. aureus* was periodically detected (2.6 log cfu/g), while *L. monocytogenes* and *Salmonella* were not detected.	(74)
Tinko dried meat	Bacteria: *S. aureus, Micrococcus luteus, Neisseria* sp., and *Acinetobacter* sp. Fungi: *Aspergillus niger, Penicillium* sp., *Rhizopus* sp., and *Aspergillus flavus*	Storage (rainy season) Cupboard Refrigerator	Almost all except for *Neisseria* sp. bacterial isolates showed potentially resistance to antibiotics used. Increased bacterial counts were observed in the cupboard-stored (2.5 × 107–3.3 × 107 cfu/ml) samples as compared to refrigerated-stored (2.0 × 107–1.4 × 107 cfu/ml).	(90)
Spanish cured meat	Mesophilic aerobic bacteria and psychrotrophic bacteria *Enterobacteriaceae, S. aureus Salmonella* spp., and *L. monocytogenes*	Commercial packaging of variety cured meat products	Non-significant detection of *Enterobacteriaceae* was observed, while *Salmonella* spp. and *L. monocytogenes* were non-detected before enrichment *S. aureus* was detected in all samples (range 3.91–4.57 log cfu/g) in considerably safe level for food safety quality	(91)
Kitoza meat	Lactic acid bacteria and coagulase-negative staphylococci *L. monocytogenes, S. aureus*	Different local manufacturer for sun-dried and/or smoked Kitoza	No *Salmonella* spp. were detected, on 1 sample contaminated with *L. monocytogenes.* *B. cereus* and *C. perfringens* under the detection threshold (2 and 1 log cfu/g, respectively). *S. aureus* has been observed to be significantly different for dried and smoke kitoza (*P* ≤ 0.001); *S. aureus* counts in dried kitoza (3.4 ± 1.0 log cfu/g) and smoked kitoza (2.3 ± 0.7 log cfu/g).	(87)
El-Guedid dried meat	LAB and coagulase negative *staphylococci* *L. mesenteroides, L. sakei*, and *S. saprophyticus* *S. aureus*	Raw meat from different origins and downstream processes (365 days storage)	*Enterobacteria* counts were observed about 2.0 ± 1.0 log cfu/g throughout the storage. *Listeria*, *Salmonella*, and anaerobic sulfite-reducing bacteria (SRA) were not detected in this study. *S. aureus* was only detected in few samples and became below the detection threshold after (<10 log cfu/g). Yeasts and molds contaminated all samples of sliced fresh meat at average level of 3.0 ± 0.7 log cfu/g and across the storage period, it became under the detection threshold.	(92)

The influence of storage on microbiological growth of Tinko dried meat samples from Ilorin, Nigeria was also studied for its related microorganism profile during the rainy season (90). The meat samples were stored in two different conditions (cupboard and refrigerator) for 5 weeks before all samples were measured for their moisture content and tested for microorganism sensitivity via the disc diffusion method. The moisture content for cupboard storage showed an increase from 35 to 65%, while refrigerated samples showed only a 50 to 55% increase in moisture content. There were four bacterial isolates (*S. aureus*, *Micrococcus luteus*, *Neisseria* sp., and *Acinetobacter* sp.) were recognized based on the colonial, cellular morphological and biochemical profile, while four fungal isolates (*Aspergillus niger*, *Penicillium* sp., *Rhizopus* sp., and *Aspergillus flavus*) were identified via colonial morphology and microscopic examination. The study has found that most of the isolated bacteria showed resistance to tested antibiotics except for the *Neisseria* sp. that was most susceptible. The bacteria count for the dried meat samples in cupboard storage elevated from 2.5 × 107 cfu/ml to 3.3 × 107 cfu/ml, while the refrigerated samples showed a reduced bacterial count from 2.0 × 107 to 1.4 × 107 cfu/ml. This has thus postulated that the storage conditions contribute to the microorganism profile of the dried meat and meat products. Among all the identified microorganisms, even though *S. aureus* is the most common food contaminant, fungal *Aspergillus* has also been highly associated with many disease developments.

Another study has appraised the consequence of commercial packaging on the microbiological activities of Spanish cured meat products (91). In this study, a variety of cured meat samples was used, and the microbiological properties were evaluated. The results have found that all samples have mean for pH values ranging from 5.79 to 5.83 and the <0.90 a_w_ values. The microbial count for most of the cured meat samples showed relative representative values as the previous study for these types of meat products, as such the mesophilic aerobic bacteria and psychotropic bacteria counted for about 4.47–7.61 log cfu/g (93). The results have also found that in at least more than half of the samples, no *Enterobacteriaceae* were detected, which is statistically significant (*p* < 0.05), and while no *Clostridium botulinum* was detected at all. Above all, it was also found that the occurrence of *S. aureus* is more common, but for *Salmonella* spp. and *L. monocytogenes*, only detected after enrichment treatment. Thus, the author has concluded that the packaging has been given a significant influence on the presence of the microorganism. This study has found that all identified microorganisms are present at a safe level, as such *S. aureus* needs more than 105 cfu/g to cause food-borne illness and for *Salmonella* spp. threshold is 107–109 cfu/g.

The traditional meat product of Malagasy or the Kitoza is a sun-dried and smoked meat strip that is important in the native diet. A study has investigated the microbial properties of the Kitoza samples obtained from various local productions in Madagascar (87). It was found that, in most of the samples, the two most dominant populations are the LAB and coagulase-negative staphylococci, with average microbial count ranging from 6 to 7 log cfu/g. Despite obtaining the samples from the local manufacturer with subpar hygienic practices, no common *Salmonella*, *Clostridium perfringens*, and *Bacillus cereus* contamination were observed, with a very rare occurrence of *L. monocytogenes.* However, *S. aureus* has been found routinely higher in salted-dried meat products as compared to salted-smoked products. The moisture, protein, fat and salt content were observed to vary on average, with higher moisture content were determined in smoked kitoza than the dried ones. Even at ambient room temperature storage, smoked kitoza has an increasing Benzo(a)pyrene content, that is fairly exceeding the limit of regulation. Therefore, alternatives such as introducing bio-protective cultures or reducing the pH value were proposed to enhance the stability of the microbial profile of kitoza. Both *Enterobacteriaceae* and *E. coli* are common indicators of fecal contamination, but in this study, both are found to be on the safe level in all tested samples, even though the counts are higher in the dried kitoza samples.

Other than that, the microbiological properties of El-Guedid, a traditional dried meat product from Algerian, were studied to describe its differences in microbiological properties as it was harbored from different origins and downstream processes (92). The results have observed that, the samples were categorized by reduced moisture content (15.6–16.3%) and a_w_ values (0.66–0.68), as well as the salt content ranging between 8.8 and 19.3% and oscillated pH values from 6.4–6.4 to 5.2–5.5. In those conditions, the microbial profile showed that there is no *Listeria* and *Salmonella* were detected, but sporadic growth of *S. aureus* was observed shortly after ripening. In the preparation of dried meat, ripening stage occurs when the product is partially dried and its typical aroma and taste characteristics started to develop due to the biochemical changes (94). In addition, the profile has also found that the LAB and coagulase-negative *Staphylococci*, with identified species *L. mesenteroides*, *Lactobacillus sakei*, and *S. saprophyticus* were predominantly in the El-Guedid samples. As these microorganisms were found throughout the processes, the populations were observed to decrease to as low as 2 log cfu/g toward the end of 365 storage. Therefore, it was suggested that an immediate or drastic drying of El-Guedid will allow the safe production of the traditional meat products. The dominantly present *L. mesenteroides* and *L. sakei* observed in this study are relevant as both are common microbial that were found in fresh packaged meat products and are responsible as the spoilage agent.

## Biochemical characteristics and changes during meat drying

**FIGURE 5:**
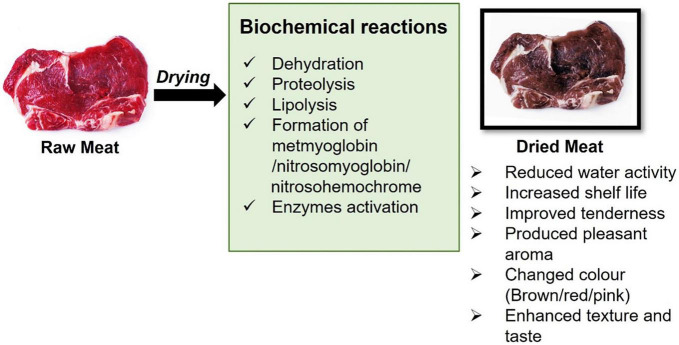
Biochemical reactions and changes occur during meat drying.

The component of meat is 75% of water, 19% of protein, 2.5% of lipid, 1.2% of carbohydrate, and 1.65% nitrogen compound (95). Meat is considered a complete source of protein (96). It also contains B complex vitamins, vitamin A and minerals such as zinc, iron, selenium, sodium, copper, magnesium, calcium, potassium, and phosphorus (97). Lipids are the most variable compounds in meats and the values can vary between 1 and 15% (98). The main component of meat composition is summarized in Supplementary Table 1. Different methods in drying meats such as dry aging and dry curing (99, 100) lead to complex biochemical reactions and finally affect the meat quality such as tenderness, juiciness, color, aroma, and flavor (101–103). The biochemical reactions and changes that occur during meat drying are summarized in Figure 5.

### Water

There are three types of water in meat which are bound, immobilized, and free (104). Bound water represents about 4–5% of water in muscle. It is held tightly by myofibrillar protein charges. Bound water cannot move among water compartments and remains unfrozen at −40^°^C. It can only be eliminated by severe drying. Immobilized water is the largest proportion of water bound in meat (around 35–75%). It is retained in the muscle ultrastructure by either steric effects or by attraction to bound water. This type of water can be removed by conventional heating and converted into ice during freezing. Meat that contains more immobilized water will have more ability to retain moisture and thus increase yields. Meanwhile, free water is held in the meat by weak capillary force and flows from the meat are not impeded (23).

A protein called myofibrils possesses a predominant role in the water holding capacity (WHC) of meat. WHC is the ability of meat to retain its inherent water during force application and/or processing (grinding, curing, thermal processing, etc.) and the water added during meat product manufacturing. Dried meat products have low water a_w_, usually reported between 0.60 and 0.80 (105). During the meat drying process, dehydration occurs in two distinct stages. In the first stage, the drying rate remains constant and equal to that of evaporation from a free liquid surface. After this stage, there is a sudden drop in the drying rate at the end of the constant-rate period, where the drying rate decreases dramatically, due to moisture diffusion being reduced by physical or chemical interactions within the food. This sudden fall in drying rate is caused by the physicochemically bound water (106). This inflection point is frequently referred to as the critical moisture content (107). The second stage of the drying period is known as the falling-rate drying period. It begins at the inflection point and extends to the final moisture content (106). Meat drying is beneficial because it can prolong its shelf-life by reducing moisture content (21).

### Proteins

Proteins in meats can be classified into three classes. They are myofibrillar, sarcoplasmic, and connective tissue. Myofibrillar plays a role in muscle contraction-relaxation and the most abundant myofibrillar is myosin. Sarcoplasm is a cytoplasm of a muscle cell and includes enzymes, intermediate, and product molecules involved in many metabolic reactions. Meanwhile, connective tissue provides support to organs and other tissues. The primary protein in connective tissue is collagen and elastin.

Meat tenderness is the most acceptable quality trait for consumers. The tenderness depends on four main components: the extent of myofibrillar protein degradation (proteolysis), sarcomere length, and the content of connective tissue (108), and endogenous proteolytic enzymes (109). Dry aging of beef *Longissimus Dorsi* was reported to cause an increase in myofibrillar degradation and a decrease in shear force compared to commercial beef (110). Degradation of myofibril by natural enzymes will improve meat tenderness (111). The changes in dry-cured meat are mostly contributed by several muscle enzymes, namely proteases and lipases, which are involved in proteolysis and lipolysis. Proteolysis contributes to changes in the texture (109), taste, and aroma (112). The first stage of proteolysis is a breakdown of major myofibrillar proteins by calpains and cathepsins. Second, generation of polypeptides this will act as substrates for peptidases to generate small peptides. Finally, there will be an intense generation of free amino acids by aminopeptidases (113). Lipolysis forms another important group of enzymatic reactions related to final sensory quality, especially in the aroma of hams (43). In lipolysis, lipases will initiate the breakdown of tri-acylglycerols and phospholipids, followed by phospholipases. Next, oxidative reactions preceded, in which aroma volatile compounds will be produced (114).

Meat color is dependent on many animal factors (species, breed, sex, age, diet, and activity) and muscle type (115). These factors both rely on the main sarcoplasmic protein, the myoglobin (23). Myoglobin is a globular protein, and it contains a non-protein portion called heme as a tightly bound prosthetic group (116). Heme is a complex of protoporphyrin IX and ferrous iron (Fe^2+^). The iron is located in the center of the heme molecule. The iron can accept six electrons in its outer orbit and can thus form six-coordinate bonds. A notable exception is two histidine residues; one proximal histidine binds directly to Fe^2+^ of the heme while the second distal histidine does not directly interact with the heme but helps stabilize the binding of O_2_ to Fe^2+^. The iron in the heme ring can exist in a reduced (ferrous/Fe^2+^) or oxidized (ferric/Fe^3+^) form. The state of iron in heme determines the color of meat. There are four forms of myoglobin in meats; oxymyoglobin (OxyMb, Fe^2+^), metmyoglobin (MetMb, Fe^3+^), deoxymyoglobin (DeoxyMb, Fe^2+^), and carboxymyoglobin (COMb, Fe^2+^) (117). OxyMb and COMb exhibit attractive and bright cherry-red color (118). DeoxyMb gives purplish-red color to the meat. Myoglobin has a greater affinity to carbon monoxide than oxygen, resulting in the increased stability of bright cherry-red COMb. Meat discoloration to brown color is seen in MetMb form where three ferrous is oxidized to a ferric state. Dried meat has a brown color (119) which indicates iron in heme is oxidized to ferric a state and formed MetMb. Salts that are added during the drying process also will lead to the darkening effect. Nitrate or nitrites added in the dry-cured meat method will react with myoglobin to produce nitrosomyoglobin, which is red in color. Nitrosomyoglobin is converted to nitrosohemochrome (pink color) when heated (119).

Meat texture profiles include hardness, cohesiveness, springiness, gumminess, and chewiness. The hardness, gumminess, and chewiness of meat are mainly influenced by soluble collagen content, fat content, and moisture content. Collagen is the most abundant protein in connective tissue and plays a role as a determinant of textural differences in the meat (120), meanwhile, elastin and reticulin are found in much smaller amounts and do not appear to contribute to unfavorable tenderness (121). Increased collagen concentration and mature crosslinks have an additive effect on the toughness of the meat and produce less tender meats. One study of dried-aged beef reported a decrease in the humidity in the first 21 days. This allows the concentration of fat, total protein, and total collagen. During the second part of the dry-aging process (14–35 days), the meat pH value increased from 5.49 to 5.66, and this value favored the water-holding capacity (by 37.33%). Meat’s enzymes (calpain, cathepsin, and collagenase) are activated and this improves the solubilization of meat proteins and collagen thus, meat texture is enhanced (120). Other authors stated that the concentration of protein and fat content increases insignificantly due to the decrease in moisture content (122, 123). Another study reported that there were no major differences between the amount of total collagen and soluble collagen during beef dry-aging (loin muscle), possibly because the study was performed on dry-aged beef for a shorter time (14 days) (124). Therefore, the total collagen content was not significantly affected by the dry-aging process. The content of soluble proteins and soluble collagen in meat could be influenced by other factors related to species and animal (within the same species or the same animal: age, sex, race, fattening status); factors related to the type of muscle considered, type II (rapid contraction/glycolytic metabolism) and type I (slow contraction/oxidative metabolism) (125).

### Lipids

Lipids are responsible for the characteristics of meats and meats products, such as aroma and flavor (126). Lipids in meats are mainly composed of triglycerides and phospholipids. Triglyceride is made up of three fatty acids and glycerol. In meat, the total content of saturated fatty acid (MUFA) (SFA) is 30–50%, while 35–50% is a monounsaturated fatty acid (MUFA) and 2–30% is polyunsaturated fatty acid (PUFA). The chemical composition in meat, such as fat content and fatty acids, causes it to become highly prone to oxidative reactions. Several researchers stated that phospholipids are essential in the development of lipid oxidation (127) because phospholipids have a higher amount of polyunsaturated fatty acids (PUFA) than triglycerides (77).

There are three main ways for lipid peroxidation: autoxidation, enzyme-catalyzed oxidation, and photo-oxidation. Autoxidation is the most important process of lipid oxidation in meat (128). Briefly, autoxidation occurs when unsaturated fatty acids and oxygen interact and therefore produce an oxidative deterioration of meat and meat products (129). Autoxidation involves three stages: initiation, propagation, and termination. In the initiation stage, an initiator (such as a hydroxyl radical) will begin the chain reaction. It extracts a hydrogen atom from the polyunsaturated fatty acid in membrane lipid and forms lipid radical. The chain reaction is then propagated when O_2_ reacts with lipid radical to form lipid peroxy radical and lipid peroxide. In the termination stage, the radical reaction stops when two radicals react and produce a non-radical species. The termination stage also can be sped up by neutralizing free radicals with the help of antioxidants such as vitamin E and vitamin C. Termination reactions are always inefficient and lead to the formation of new reactive compounds. The mechanism that ensures termination efficiently is the decomposition of peroxy and alkoxy radicals to give rise to secondary products such as alkanes, alcohols, and carbonyl compounds (127).

Raw meat has weak flavored, salty, metallic, and rare (bloody) with a slightly sweet aroma (130). Aroma generation in dry meat is through several reactions such as microbial fermentation of carbohydrates, enzymatic hydrolysis of proteins, Maillard reactions (furans, sulfur, and nitrogen compounds), and lipid peroxidation (131). However, in this section, we will focus on the aroma of meat generated through lipid peroxidation. Lipid oxidation generally has negative effects on meats and meats products. But, in terms of aroma, it contributes to the pleasant aroma (132, 133). In dry fermented sausages, lipid autoxidation produces volatile and non-volatile compounds that have beneficial effects on odor and taste (23). The main substrates of these chemical reactions are PUFA, and the primary product of oxidation is odorless and tasteless but is very unstable and quickly decomposes into a high number of non-volatile and volatile compounds through very complex reaction pathways. These final products will enhance the taste and aroma of foods and include a large variety of compounds: aldehydes, ketones, alkanes, alcohols, esters, furanes, and carboxylic acids. The addition of some ingredients and additives such as nitrite and ascorbate will affect lipid oxidation in dry fermented sausages (134).

In dried-cured meat, high salt content and low aw contribute to lipid hydrolysis. Muscle phospholipases are responsible for meat lipolysis while neutral lipases hydrolyze triglycerides in adipose tissue to mono-and diglycerides and free fatty acids. PUFA hydrolysis is preferred and triglycerides containing oleic and oleic acids are more hydrolyzed than those rich in SFA. The free fatty acids that are highly released up to 10 months of ham processing will undergo lipid autoxidation which mainly degrades PUFA and MUFA, while better stability in SFA causes its accumulation. Production of volatile compounds by these reactions are important for the final aroma of dry-cured meat (135). However, meat with a high level of PUFA can rapidly oxidize and cause color deterioration and rancidity (23).

### Carbohydrates

Meat is a muscle part of an animal that underwent complex chemical and biochemical processes (136). In living muscle tissue, carbohydrates are present in a small concentration. The most predominant form of carbohydrate in the muscle is glycogen (137). Glycogen is a branched-chain polysaccharide made from α-D glucose. The glucose residues are linked linearly by α-1,4 glycosidic bonds and after 8–14 glucosyl residues, a chain of glucose residues branches off via α-1,6 glycosidic bonds (138). Muscle glycogen functions as an energy store to supply energy for muscle contraction through aerobic glycolysis. Aerobic glycolysis requires oxygen, therefore after death aerobic pathway stops. To maintain ATP production needed for membrane ion pumps and cell integrity, glycogen is degraded to pyruvate through anaerobic glycolysis. The final product of anaerobic glycolysis, lactic acid will accumulate causing acidification and hardening of the meats.

Glycogen has been studied for its role in the determination of pHu (ultimate pH) and meat quality (139). Glycogen concentration will drop to less than 1% at 24 h post-mortem. However, glycogen is depleted in animals exposed to long-term pre-slaughter stress. Glycogen depletion (less than 0.6%) will hinder normal post-mortem pH decline. The other carbohydrates are glucose, other mono- and disaccharides (0.1–0.15%), and intermediates of glycogen metabolism (140). Glycogenolysis has a major role in changes occurring in muscle tissue post-mortem and affects meat quality. Two enzymes involved in glycogenolysis are glycogen phosphorylase and debranching enzymes. A study reported that residual glycogen in pigs and cattle lowers the yield of meat in cooking, decreases in protein content of muscle tissue, and influences the sensory quality of the meat (141). Type IIB muscle fibers are known as fast-twitch fibers or fast glycolytic fibers and contain a large amount of glycogen (142). A rapid pH decline was observed in muscles containing a higher percentage of type IIB fibers compared to ones containing more type IB fibers (143). Meat derived from muscle rich in type IIB muscle fiber requires a longer time for the dry-aging process (144). Prolonging the dry-aging time will improve the tenderness in meat with type IIB muscle fibers (143).

### Vitamins

Meat and meat products provide a good source of most water-soluble vitamins such as vitamin B1, B2, B3, B6, and vitamin B12 except for vitamin C. The concentration of vitamin B ranges from a few micrograms to several milligrams. For fat-soluble vitamins, most of them present at low levels. Vitamin A is detected in a small amount (0–40 mg/100 g), vitamin D at 0.03–0.60 mg/100 g (cholecalciferol), and 0.4–0.20 mg/100 g (25-hydroxy vitamin D) (17). Meat is not an important source of the other fat-soluble vitamins; vitamin E and K (145, 146). Vitamins are degraded by exposure to heat, water, and sunlight. In conventional drying methods, vitamins in meats are degraded (26). However, some modern drying techniques such as freeze-drying will not cause protein denaturation or loss of vitamins (34).

### Minerals

Meat is an excellent source of minerals. Iron and zinc are the most important nutrients found mainly in red meat, with 100 g providing at least one-quarter of daily adult requirements (147). Other minerals present in meats are phosphorus, potassium, magnesium, copper, selenium, calcium, and sodium (23). The iron levels remain unchanged even after the high temperature is applied to ostrich, beef, and horse meats (148). In another study, beef jerky dried at 60°C showed an increase in iron content (149). However, in ostrich dried meat, the addition of salt followed by spices significantly decreased the heme iron by 18% (2).

## Safety and challenges of dried meats and how to prevent them

**FIGURE 6:**
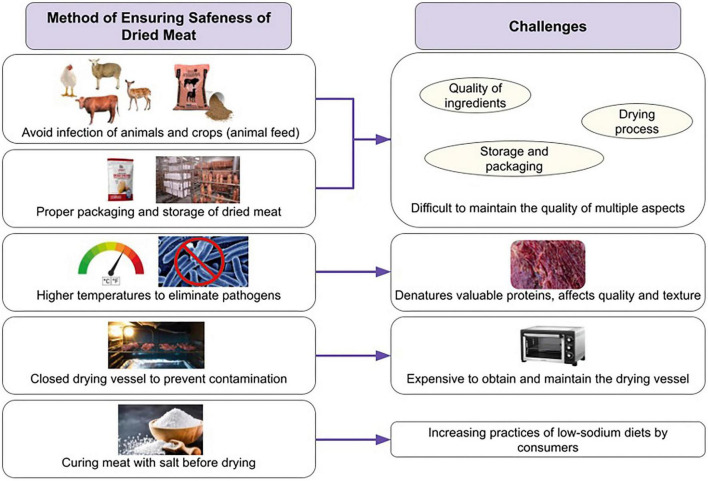
Methods of ensuring the safeness of dried meat along with their challenges.

While dried meat has long been deemed reasonably safe in the market, there are still some concerns involving the microbial growth and contents of the dried meat, not to mention certain challenges in the production process. Thus, precautions are taken to ensure that the safety of the consumers is prioritized (Figure 6). Although the dehydration of meat reduces the contamination of microbes in dried meat, outbreaks of gastroenteritis caused by consumption of dried meat have been reported in the past. *S. aureus* and *Salmonella* contaminations in meat jerky were the main causes of the major outbreaks of illnesses in New Mexico in 1966 and 1995 (150, 151). The outbreaks occurred due to insufficient temperatures during the drying process, as the bacteria were not destroyed nor removed from the dried meat products (151). In another case in Oregon, *E. coli* contamination in dried meat also affected the health of 11 people back in 1995, with the meat being deer meat (151). Interestingly, the cause of the contamination was also found to be drying temperatures insufficient to kill the bacteria. Moreover, it was also stated that an experiment involving the dehydration of meat at 62.8°C for 10 h failed to eliminate the *E. coli* O157:H7 strain that was inoculated into the jerky meat. That being said, dried meat is still susceptible to bacterial contamination, although the water content in the meats has been reduced. This makes it concerning for consumers as something as simple as improper packaging could cause dried meat to become a safety hazard.

Due to its contact with the ambient microbiota during processing, drying, and point of sale, dried meat is also prone to infection by fungi, which may in turn release mycotoxins. A study concluded that mycotoxins could be found in dried beef samples from Nigerian regions Igede and Ikole, in which mycotoxins are frequently linked to serious health issues (152). It was mentioned that continuous exposure to mycotoxins like cyclopiazonic acid (CPA), alpha zearalenone, aflatoxin, ochratoxin A, sterigmatocystin and tenuazonic acid could cause immunotoxic, hepatotoxic, and carcinogenic effects on multiple organs in the human body (152). It was also discovered that certain mycotoxins’ immune-modulating properties exacerbate the negative health impacts of serious illnesses that affect Africa, such as malaria, kwashiorkor, and acquired immunodeficiency syndrome (AIDS) (153). Mycotoxins are secondary metabolites produced by fungi that mostly infect crops (154).

It was speculated that mycotoxins probably contaminated the dried beef samples through contaminated feed and urged further studies in this area for the sake of consumers’ health (152). From this, it can be said that the health of the animals directly influences the safety of consuming dried meat. Among animals, pigs are thought to be the most susceptible agricultural animals to mycotoxins, whereas ruminants like sheep and cattle are less vulnerable since their rumen fluid can break these mycotoxins down into less hazardous metabolites (154). While dried meat is supposedly produced to dehydrate meats, certain conditions throughout the drying, storing, and commercial process of the products may favor the growth of mycotoxin-producing fungi. Since fungi are generally found in humid places with adequate nutrients (155), inconsistencies in dried meat processing can cause contamination by mycotoxins. For instance, humid storage places and faulty packaging of dried meat would rehydrate the product and make it favorable for fungi growth. As such, mycotoxins continue to be a serious problem in food safety as both the feed of the animals and the animals themselves need to be protected from fungi infection. This poses as a huge challenge for the dried meat industry as multiple aspects in the production of dried meat need to be taken care of to ensure that the dried meat is safe from mycotoxins.

Apart from that, adulteration is also an issue to be noticed in the dried meat industry. A study concluded that not only is soya not found in all of the dried meat samples, only very low amounts of either declared or undeclared gluten were found in dried meat as well (156). Additionally, the study also stated that dried meat is less likely to contain undeclared pork compared to other kinds of meat products. This, however, does not omit the fact that small amounts of undeclared constituents can be present in dried meat, especially in terms of sensitivity to ethics, allergies, economy and religion (156). Of course, there are precautions that can be made to ensure that dried meat products are safe to be consumed and enjoyed. As discussed previously, the failure to eliminate bacteria in dried meat due to insufficient temperatures in drying processes can be improved by increasing the drying temperatures. As an example, it was stated that 63°C is recommended for drying beef and lamb while 74°C should be used for drying poultry (151). As for venison, it was suggested to pre-cook the meat to 74°C before the drying process begins (151). These temperatures can be seen as the minimum limits which would suffice in eliminating pathogens from the dried meat products, thus removing the health hazards in dried meat (Supplementary Table 2). However, high temperatures could also denature valuable proteins that contribute to the texture and quality of the dried meat itself, as discussed previously. Ethically, the safety of consumers should be prioritized, but this dilemma is nonetheless a challenge for the dried meat industry to balance.

Aside from that, the method of drying itself should also be taken into consideration when ensuring the safety of dried meat. Multiple articles have mentioned that sun-drying meat increases the risk of contamination in the products since it is unprotected and exposed to an environment where insects, pathogens and other weatherly factors could affect the meat itself (40, 151). Thus, if the dried meat were to require higher temperatures, a closed vessel should be used to ensure that the meat is dried in a relatively uncontaminated environment. The issue with this safety precaution is that convection vessels might be overbudget for small businesses in the dried meat field and increasing the price of dried meat products to cover the costs might reduce the demand of dried meat (Figure 6). From this perspective, the fact that open sun drying is still commonly used to dry meat is unfortunate but understandable. Curing the meat before drying it also has protective properties against microbial contamination of dried meat. It was found that dried beef that was cured prior to its drying had less *E. coli* O157:H7 after being processed (157). Curing the meat with salt, sugar, and sodium nitrite increases the lethality of pathogens like *E. coli* O157:H7, *L. monocytogenes*, and *Salmonella* (151). From this, cured dried meat could be considered if safety in the products is prioritized. However, the demands of the market might lean toward lower sodium contents in dried meat as consumers nowadays are more aware of the health benefits of low sodium diets (158). Nevertheless, it was said that neither of the curing ingredients could effectively increase pathogen lethality by themselves (151). With that, it might be better to utilize multiple curing ingredients at the same time to ensure the safety of dried meat in the market. Overall, dried meat can be safe to be consumed as long as the necessary steps are taken to avoid contamination of the product. Relevant regulations are in place to ensure that dried meat productions follow the required precautionary steps to prevent unnecessary illnesses.

## Advanced approaches in identifying the quality of dried meat and meat products

Recent trends in the progress of drying meat and meat products are related to consumer needs, including nutrition, natural products, human wellbeing, safety, and health concerns. Hence, novel strategies have been developed and implemented in processing, preparation, production, storage, and distribution systems to induce quantitative and qualitative changes in dried meat product composition and to optimize the health benefits to the consumers. Advanced technology includes the utilization of omics approaches, particularly genomics, transcriptomics, proteomics, and metabolomics. These approaches are useful in determining the drying meat and meat products quality, as well as providing crucial information on the products. The application of omics technology could enhance knowledge on the expression of genes, proteins, and metabolites (hydrophilic and lipophilic), metabolic and biochemical processes, and underlying candidate biomarkers to assess meat quality, despite the various animal species, breeds, genders, and quality trait of the dried meat source. Next-generation sequencing (NGS) could be used for the certification of mixed meat products. There are many omics done on the meat quality. However, there is a lack of research on dried meat. Further studies warrant to be conducted on dried meat by omics technology to evaluate the quality and safety aspects.

## Conclusion and future perspectives

Dried meat is processed from fresh meat because it can prolong the shelf-life and give unique flavors. Various types of dried meats can be found all around the world, each has different combinations of techniques to achieve its own distinctive sensory profiles. The dried meat can be categorized based on its geographical origin or processing techniques. Similarly, the health benefits, quality and overall contents of the dried meat depend on the type of meat, presence of spices and drying process involved in its production. This of course also influences the taste and preference of the market as well. As an addition, emphasizing the health benefits of dried meat is vital for attracting consumers that are more health conscious. Hence, dried meats can also be rich in micro and macronutrients, with good quality, as compared to fresh meat.

The drying process in production of dried meat products is important. This is because it can influence the flavor profiles, texture and retained nutritional value of the dried meat. Some examples of the drying techniques include convective-drying, sun-drying, freeze-drying and air-drying. The combination of suitable drying methods and proper drying conditions, which is largely based on the types of meat, is vital for the quality and longevity of the dried meat. Parameters such as temperature, drying period, relative humidity and moisture contents should be optimized to ensure the efficiency of the drying process. Therefore, several advanced drying techniques have been developed and studied extensively, such as ultrasound-assisted vacuum drying and freeze drying. These methods have better implications for the dried meat, in terms of efficient drying and shelf life, while conserving the quality and sensory aspects of the dried meat.

Non-meat ingredients are added during the process to improve the quality of the dried meat. Generally, salt, spice, herbs and other additives such as preservatives and coloring are added in the process of making dried meat and meat products for several reasons. The reasons include adding flavor, better sensory qualities, antimicrobial properties, health benefits and better food safety. Regardless, optimization on the amount of each ingredient can be done depending on the type of dried meats to guarantee the quality and safety of the products. Furthermore, the spices and herbs also add value to the dried meat, especially in terms of health benefits, sensory qualities and shelf life. Nonetheless, the demands for organic and natural dried meats have increased significantly among consumers in recent years (61). Thus, new combinations of non-meat ingredients, especially spices, herbs and plant extracts are currently being studied to substitute conventional additives to satisfy the demand. These natural preservatives include garlic, thyme, rosemary, chestnut, sage, cranberry, oregano and grapeseed, to satisfy the demand (60).

The physicochemical and microbiological characteristics are used to assess the quality and safety of dried meat and meat products. pH, water activity, a_w_, lipid oxidation, proximate composition, color and sensory characteristics are the parameters discussed in this review article. Meanwhile, the microbiological characteristics of dried meat focused on various factors that influenced the microbiological stability of the product, which include pH, drying method, storage conditions, to name a few. Common microorganisms that are found in meat products include LAB, *Listeria* spp., *Staphylococcus* spp., *Aspergillus* spp., and *Salmonella* spp. Identifying the critical limits and optimizing each step and conditions in processing to these factors are vital to avoid microbial infestations on the final product. Thus, there should be more dried meat processing plants that implement Hazard Analysis Critical Control Point (HACCP) and Good Manufacturing Practices (GMPs) to aid the quality control and assurance of the dried meat products. It should also be noted that the processing of fresh meat into dry meat causes biochemical changes. This changes in protein, lipid, carbohydrate, vitamin and minerals of the meat. Nevertheless, there are still challenges in ensuring the quality and safety and dried meat products that need to be addressed. Application of omics approaches has been explored and studied to combat the challenges, importantly in assessing the meat characteristics. In addition, the use of Next Generation Sequencing (NGS) in evaluating the dried meat for any traces of adulteration is also an exciting path for meat analysis, which also have been highlighted in this review. Hence, only by understanding and addressing every aspect of food safety and developing new methods on quality assessment that the production of dried meat can be expanded in the market.

## Author contributions

AM and HSH: conceptualization, validation, supervision, and funding acquisition. HSH, UKA, and NAKA: investigation. UKA and AM: data curation. AM, HSH, NFM, SXYC, ERR, NHCL, NAKA, NAFA, FAJ, HB, MNS, NK, and UKA: writing original draft preparation. FAJ, HSH, and AM: writing – review and editing. All authors have read and agreed to the published version of the manuscript.

## References

[1] Dinçer EA. Dried meat products obtained by different methods from past to present. *Food Rev Int.* (2021) 2021:1–20. 10.1080/87559129.2021.1956944

[2] Zdanowska-Sasiadek Z Marchewka J Horbanczuk JO Wierzbicka A Lipinska P Józwik A Nutrients composition in fit snacks made from ostrich, beef and chicken dried meat. *Molecules.* (2018) 23:1–13. 10.3390/molecules23061267 29799493 PMC6099787

[3] Jones M Arnaud E Gouws P Hoffman LC. Processing of South African biltong - A review. *South. Afr J Anim Sci.* (2017) 47:743–57. 10.4314/sajas.v47i6.2

[4] Rao W Wang Z Shen Q Li G Song X Zhang D. LF-NMR to explore water migration and water–protein interaction of lamb meat being air-dried at 35^°^C. *Drying Technol.* (2018) 36:366–73. 10.1080/07373937.2017.1339084

[5] Hou C Kang N Schlosser C Zhao M Wang Z Zhang D. Heterocyclic aromatic amines in commercial Chinese dried meat products. *J Food Nutri Res.* (2018) 57:151–60. 10.1007/s10068-014-0284-0

[6] Nathakaranakule A Kraiwanichkul W Soponronnarit S. Comparative study of different combined superheated-steam drying techniques for chicken meat. *J Food Eng.* (2007) 80:1023–30. 10.1016/j.jfoodeng.2006.04.067

[7] Kim DH Shin D-M Lee JH Kim YJ Han SG. Effect of different brine injection levels on the drying characteristics and physicochemical properties of beef jerky. *Food Sci Anim Resour.* (2022) 42:98–110. 10.5851/kosfa.2021.e66 35028577 PMC8728507

[8] Kaban G. Sucuk and pastirma: microbiological changes and formation of volatile compounds. *Meat Sci.* (2013) 95:912–8. 10.1016/j.meatsci.2013.03.021 23608196

[9] Chabbouh M Sahli A Bellagha S. Does the spicing step affect the quality and drying behaviour of traditional kaddid, a Tunisian cured meat? *J Sci Food Agric.* (2013) 93:3634–41. 10.1002/jsfa.6319 23893302

[10] Gagaoua M Boudechicha HR. Ethnic meat products of the North African and Mediterranean countries: An overview. *J. Ethn. Foods.* (2018) 5:83–98. 10.1016/j.jef.2018.02.004

[11] Gutiérrez NO Caro I Mateo J. Dry-Cured Cecina. In *Production of Traditional Mediterranean Meat Products.* Berlin: Springer (2022). p. 87–94. 10.1007/978-1-0716-2103-5_10

[12] Lorenzo JM Fonseca S Gómez M Domínguez R. Influence of the salting time on physico-chemical parameters, lipolysis and proteolysis of dry-cured foal “cecina”. *LWT - Food Sci Technol.* (2015) 60:332–8. 10.1016/j.lwt.2014.07.023

[13] Aristoy MC Mora L Toldrá F. Histidine-containing dipeptides: Properties and occurrence in foods. In: *Encyclopedia of Food and Health.* Oxford: Academic press (2016). 338–42. 10.1016/B978-0-12-384947-2.00777-7

[14] Fleming JA Kris-Etherton PM. The evidence for α-Linolenic acid and cardiovascular disease benefits: Comparisons with eicosapentaenoic acid and docosahexaenoic acid. *Adv Nutri.* (2014) 5:863S–76S. 10.3945/an.114.005850 25398754 PMC4224228

[15] Kris-Etherton PM Innis S Assocition AD. Position of the american dietetic association and dietitians of canada: dietary fatty acids. *J Am Diet Assoc.* (2007) 107:1599–611. 10.1016/j.jada.2007.07.024 17936958

[16] Jasińska K Kurek A. The effect of oil plants supplementation in pig diet on quality and nutritive value of pork meat. *Anim Sci Pap Rep.* (2017) 35:137–46.

[17] Djinovic-Stojanovic JM Nikolic DM Vranic DV Babic JA Milijasevic MP Pezo LL Zinc and magnesium in different types of meat and meat products from the Serbian market. *J Food Composit Analy.* (2017) 59:50–4. 10.1016/j.jfca.2017.02.009

[18] Santos HO Teixeira FJ Schoenfeld BJ. Dietary vs. pharmacological doses of zinc: A clinical review. *Clin Nutri.* (2020) 39:1345–53. 10.1016/j.clnu.2019.06.024 31303527

[19] Uwitonze AM Razzaque MS. Role of magnesium in vitamin d activation and function. *J Am Osteopath Assoc.* (2018) 118:181–9. 10.7556/jaoa.2018.037 29480918

[20] Šuput D Lazić V Pezo L Gubić J Šojić B Plavšić D Shelf life and quality of dehydrated meat packed in edible coating under modified atmosphere. *Rom Biotechnol Lett.* (2019) 24:545–53. 10.25083/rbl/24.3/545.553

[21] Mishra B Mishra J Pati P Rath P. Dehydrated meat products: A review. *Int J Livestock Res.* (2017) 7:10–22. 10.5455/ijlr.20170812035616

[22] Moses J Norton T Alagusundaram K Tiwari B. Novel drying techniques for the food industry. *Food Eng Rev.* (2014) 6:43–55. 10.1007/s12393-014-9078-7

[23] Cobos A Diaz O. Chemical composition of meat and meat products. In: Cheung P editor. *Handbook of Food Chemistry.* Berlin: Springer (2014). p. 1–32. 10.1007/978-3-642-41609-5_6-1

[24] Huang T Nip W. Intermediate-moisture meat and dehydrated meat. In *Meat Science and Applications.* Boca Raton, FL: CRC Press (2001). p. 403–42.

[25] Rahman MS Labuza TP. Water activity and food preservation. In: Rahman MS editor. *Handbook of Food Preservation.* Boca Raton, FL: CRC Press (2007). p. 465–94.

[26] Doymaz İ Karasu S Baslar M. Effects of infrared heating on drying kinetics, antioxidant activity, phenolic content, and color of jujube fruit. *J Food Meas Charact.* (2016) 10:283–91. 10.1007/s11694-016-9305-4

[27] Laopoolkit P Suwannaporn P. Effect of pretreatments and vacuum drying on instant dried pork process optimization. *Meat Sci.* (2011) 88:553–8. 10.1016/j.meatsci.2011.02.011 21396788

[28] Koc B Eren I Ertekin FK. Modelling bulk density, porosity and shrinkage of quince during drying: The effect of drying method. *J Food Eng.* (2008) 85:340–9. 10.1016/j.jfoodeng.2007.07.030

[29] Aksoy A Karasu S Akcicek A Kayacan S. Effects of different drying methods on drying kinetics, microstructure, color, and the rehydration ratio of minced meat. *Foods.* (2019) 8:216. 10.3390/foods8060216 31216705 PMC6617532

[30] Tekin ZH Baslar M. The effect of ultrasound-assisted vacuum drying on the drying rate and quality of red peppers. *J Therm Anal Calorim.* (2018) 132:1131–43. 10.1007/s10973-018-6991-7

[31] Chibuzo NS Osinachi UF James MT Chigozie OF Dereje B Irene CE. Technological advancements in the drying of fruits and vegetables: A review. *Afr J Food Sci.* (2021) 15:367–79. 10.5897/AJFS2021.2113

[32] Tao Y Sun D-W. Enhancement of food processes by ultrasound: A review. *Crit Rev Food Sci Nutr.* (2015) 55:570–94. 10.1080/10408398.2012.667849 24915392

[33] Liu Y Zhang Z Hu L. High efficient freeze-drying technology in food industry. *Crit Rev Food Sci Nutr.* (2022) 62:3370–88. 10.1080/10408398.2020.1865261 33393368

[34] Ma Y Wu X Zhang Q Giovanni V Meng X. Key composition optimization of meat processed protein source by vacuum freeze-drying technology. *Saudi J Biol Sci.* (2018) 25:724–32. 10.1016/j.sjbs.2017.09.013 29740237 PMC5936978

[35] Berk Z. Freeze drying (lyophilization) and freeze concentration. In *Food Process Engineering and Technology.* San Diego, CA: Academic Press (2013). 567–81. 10.1016/b978-0-12-415923-5.00023-x

[36] Sagar VR Suresh KP. Recent advances in drying and dehydration of fruits and vegetables: A review. *J Food Sci Technol.* (2010) 47:15–26. 10.1007/s13197-010-0010-8 23572596 PMC3550996

[37] Bas̨lar M Kiliçli M Toker OS Saʇdiç O Arici M. Ultrasonic vacuum drying technique as a novel process for shortening the drying period for beef and chicken meats. *Innov Food Sci Emerg Technol.* (2014) 26:182–90. 10.1016/j.ifset.2014.06.008

[38] Xu L Fang X Wu W Chen H Mu H Gao H. Effects of high-temperature pre-drying on the quality of air-dried shiitake mushrooms (*Lentinula edodes*). *Food Chem.* (2019) 285:406–13. 10.1016/j.foodchem.2019.01.179 30797364

[39] Kilic A. Low temperature and high velocity (LTHV) application in drying: Characteristics and effects on the fish quality. *J Food Eng.* (2009) 91:173–82. 10.1016/j.jfoodeng.2008.08.023

[40] Ayanwale B Ocheme O Oo O. The effect of sun-drying and oven-drying on the nutritive value of meat pieces in hot humid environment. *Pakistan J Nutri.* (2007) 6:370–4. 10.3923/pjn.2007.370.374

[41] Midilli A Kucuk H. Mathematical modeling of thin layer drying of pistachio by using solar energy. *Energy Convers Manag.* (2003) 44:1111–22. 10.1016/S0196-8904(02)00099-7

[42] Choi YS Ku SK Park JD Kim HJ Jang A Kim YB. Effects of drying condition and binding agent on the quality characteristics of ground dried-pork meat products. *Korean J Food Sci Anim Resour.* (2015) 35:597–603. 10.5851/kosfa.2015.35.5.597 26761886 PMC4670887

[43] Toldrá F. The role of muscle enzymes in dry-cured meat products with different drying conditions. *Trends Food Sci Technol.* (2006) 17:164–8. 10.1016/j.tifs.2005.08.007

[44] Ba HV Hwang I Jeong D Touseef A. Principle of meat aroma flavors and future prospect. In *Latest Research into Quality Control.* London: IntechOpen (2012). 145–76. 10.5772/51110

[45] Yalçin MY S̨eker M. Effect of salt and moisture content reduction on physical and microbiological properties of salted, pressed and freeze dried turkey meat. *LWT-Food Sci Technol.* (2016) 68:153–9. 10.1016/j.lwt.2015.12.032

[46] Arnau J Gou P Comaposada J. Effect of the relative humidity of drying air during the resting period on the composition and appearance of dry-cured ham surface. *Meat Sci.* (2003) 65:1275–80. 10.1016/S0309-1740(03)00036-6 22063770

[47] Ogunsola OO Omojola AB. Qualitative evaluation of Kilishi prepared from beef and pork. *Afr J Biotechnol.* (2008) 7:1753–8. 10.5897/ajb08.354

[48] Cantalejo MJ Zouaghi F Pérez-Arnedo I. Combined effects of ozone and freeze-drying on the shelf-life of Broiler chicken meat. *LWT - Food Sci Technol.* (2016) 68:400–7. 10.1016/j.lwt.2015.12.058

[49] Bampi M Schmidt FC Laurindo JB. A fast drying method for the production of salted-and-dried meat. *Food Sci Technol.* (2019) 39:526–34. 10.1590/fst.24418

[50] Pawlak T Gawałek J Ryniecki A Stangierski J Siatkowski I Pepliñska B Microwave vacuum drying and puffing of the meat tissue – process analysis. *Drying Technol.* (2019) 37:156–63. 10.1080/07373937.2018.1444635

[51] Elmas F Bodruk A Köprüalan Ö Arıkaya S̨ Koca N Serdaroğlu FM Drying kinetics behavior of turkey breast meat in different drying methods. *J. Food Process Eng.* (2020) 43:1–11. 10.1111/jfpe.13487

[52] Dinçer EA Atlı B Çakmak Ö Canavar S Çalışkan A. Drying kinetics and quality characteristics of microwave-assisted hot air dried beef chips. *J Microw Power Electromagn Energy.* (2021) 55:219–35. 10.1080/08327823.2021.1952836

[53] Dimakopoulou-Papazoglou D Katsanidis E. Effect of maltodextrin, sodium chloride, and liquid smoke on the mass transfer kinetics and storage stability of osmotically dehydrated beef meat. *Food Bioproc Technol.* (2017) 10:2034–45. 10.1007/s11947-017-1973-5

[54] Barat JM Baigts D Aliño M Fernández FJ Pérez-García VM. Kinetics studies during NaCl and KCl pork meat brining. *J Food Eng.* (2011) 106:102–10. 10.1016/j.jfoodeng.2011.04.022

[55] Santchurn SJ Collignan A Trystram G. Impact of solute molecular mass and molality, and solution viscosity on mass transfer during immersion of meat in a complex solution. *J Food Eng.* (2007) 78:1188–201. 10.1016/j.jfoodeng.2005.12.031

[56] Dima C Dima S. Essential oils in foods: extraction, stabilization, and toxicity. *Curr Opin Food Sci.* (2015) 5:29–35. 10.1016/j.cofs.2015.07.003

[57] Karabagias IK Badeka A Kontakos S Karabournioti S Kontominas MG. Characterization and classification of *Thymus capitatus* (L.) honey according to geographical origin based on volatile compounds, physicochemical parameters and chemometrics. *Food Res Int.* (2014) 55:363–72. 10.1016/j.foodres.2013.11.032 24176380

[58] Singletary K. Black pepper: Overview of health benefits. *Nutri Today.* (2010) 45:43–7. 10.1097/NT.0b013e3181cb4539

[59] Juneja VK Valenzuela-Melendres M Heperkan D Bautista D Anderson D Hwang CA Development of a predictive model for *Salmonella* spp. reduction in meat jerky product with temperature, potassium sorbate, pH, and water activity as controlling factors. *Int J Food Microbiol.* (2016) 236:1–8. 10.1016/j.ijfoodmicro.2016.06.028 27427870

[60] Suryati T Astawan M Lioe HN Wresdiyati T Usmiati S. Nitrite residue and malonaldehyde reduction in dendeng — Indonesian dried meat — influenced by spices, curing methods and precooking preparation. *Meat Sci.* (2014) 96:1403–8. 10.1016/j.meatsci.2013.11.023 24361560

[61] Sebranek J Bacus J. Natural and organic cured meat products: Regulatory, manufacturing, marketing, quality and safety issues. *Am Meat Sci Assoc White Paper Ser.* (2007) 1:1–15.

[62] Govari M Pexara A. Nitrates and nitrites in meat products. *J Hellenic Vet Med Soc.* (2015) 66:127–40. 10.12681/jhvms.15856

[63] Chamandoost S Moradi MF Hosseini MJ. A review of nitrate and nitrite toxicity in foods. *J Hum Environ Health Promot.* (2016) 1:80–6. 10.29252/jhehp.1.2.80

[64] Peter KV Shylaja MR. Chapter 1-Introduction to herbs and spices: definitions, trade and applications. In: Peter KV editor. *Handbook of Herbs and Spices* (Second Edition). Sawston: Woodhead Publishing (2012). p. 1–24. 10.1533/9780857095671.1

[65] Nanasombat S Wimuttigosol P. Antimicrobial and antioxidant activity of spice essential oils. *Food Sci Biotechnol.* (2011) 20:45–53. 10.1007/s10068-011-0007-8

[66] Istrati D Constantin O Ionescu A Vizireanu C Dinică R. Study of the combined effect of spices and marination on beef meat vacuum packaged. Annals of the University” Dunarea de Jos” of Galati-Fascicle VI. *: Food Technol.* (2011) 35:75–85.

[67] Saeed MW Gillani SW Mahmood RK Usman M. Assessment of the antihyperlipidemic effect of garlic vs pitavastatin in patients with moderate hyperlipidemia: A metanalysis of randomized controlled trials. Chiang Mai Univer. *J Nat Sci.* (2021) 20:e2021087. 10.12982/CMUJNS.2021.087

[68] Elbahnasawy AS Valeeva ER El-Sayed EM Rakhimov II. The impact of thyme and rosemary on prevention of osteoporosis in rats. *J Nutri Metabol.* (2019) 2019:1431384. 10.1155/2019/1431384 31049223 PMC6462344

[69] Jeena K Liju VB Umadevi NP Kuttan R. Antioxidant, anti-inflammatory and antinociceptive properties of black pepper essential oil (*Piper nigrum* Linn). *J Essent Oil Bearing Plants.* (2014) 17:1–12. 10.1080/0972060X.2013.831562

[70] González-Fandos E Dominguez JL. Effect of potassium sorbate washing on the growth of Listeria monocytogenes on fresh poultry. *Food Control.* (2007) 18:842–6. 10.1016/j.foodcont.2006.04.008

[71] Jia W Wu X Li R Liu S Shi L. Effect of nisin and potassium sorbate additions on lipids and nutritional quality of Tan sheep meat. *Food Chem.* (2021) 365:130535. 10.1016/j.foodchem.2021.130535 34256226

[72] Sebranek JG Bacus JN. Cured meat products without direct addition of nitrate or nitrite: what are the issues? *Meat Sci.* (2007) 77:136–47. 10.1016/j.meatsci.2007.03.025 22061404

[73] Marco A Navarro JL Flores M. Quantitation of selected odor-active constituents in dry fermented sausages prepared with different curing salts. *J Agricul Food Chem.* (2007) 55:3058–65. 10.1021/jf0631880 17381109

[74] Petit T Caro Y Petit AS Santchurn SJ Collignan A. Physicochemical and microbiological characteristics of biltong, a traditional salted dried meat of South Africa. *Meat Sci.* (2014) 96:1313–7. 10.1016/j.meatsci.2013.11.003 24334054

[75] Lim DG Lee SS Seo KS Nam KC. Effects of different drying methods on quality traits of Hanwoo beef jerky from low-valued cuts during storage. *Korean J Food Sci Anim Resour.* (2012) 32:531–9. 10.5851/kosfa.2012.32.5.531

[76] Taormina PJ Sofos JN. Low water activity meat products. In: Gurtler J Doyle M Kornacki J editors. *The microbiological safety of low water activity foods and spices. food microbiology and food safety().* New York, NY: Springer (2014). p. 127–64. 10.1007/978-1-4939-2062-4

[77] Amaral AB Solva MVD Lannes SCDS. Lipid oxidation in meat: Mechanisms and protective factors - A review. *Food Sci Technol.* (2018) 38:1–15. 10.1590/fst.32518

[78] Ferreira VC Martins TD Batista ED Santos EP Silva FA Araujo IB Physicochemical and microbiological parameters of dried salted pork meat with different sodium chloride levels. *Food Sci Technol.* (2013) 33:382–6. 10.1590/S0101-20612013005000055

[79] Mgbemere V Akpapunam M Igene J. Effect of groundnut flour substitution on yield, quality and storage stability of kilishii – a Nigerian indigenous dried meat product. *Afr J Food Agric Nutr Dev.* (2011) 11:4718–38. 10.4314/ajfand.v11i2.65924

[80] Chukwu O Imodiboh LI. Influence of storage conditions on shelf-life of dried beef product (Kilishi). *World J Agric Sci.* (2009) 5:34–9.

[81] Lee JA Kim HY. Physicochemical properties of crust derived from dry-aged Holstein and Hanwoo loin. *J Anim Sci Biotechnol.* (2020) 62:692–701. 10.5187/jast.2020.62.5.692 33089234 PMC7553849

[82] Elmas F Bodruk A Köprüalan Ö Arıkaya S̨ Koca N Serdaroğlu FM The effect of pre-drying methods on physicochemical, textural and sensory characteristics on puff dried Turkey breast meat. *LWT.* (2021) 145:111350. 10.1016/j.lwt.2021.111350

[83] Afifah N Ratnawati L Indrianti N Sarifudin A. The effect of pre-drying treatments on the quality of dehydrated ground beef. *IOP Confer Ser Earth Environ Sci.* (2021) 924:012006. 10.1088/1755-1315/924/1/012006

[84] Oyinloye TM Yoon WB. Effect of freeze-drying on quality and grinding process of food produce: A review. *Processes.* (2020) 8:1–23. 10.3390/PR8030354

[85] Jalarama RK Pandey MC Harilal PT Radhakrishna K. Optimization and quality evaluation of freeze dried mutton manchurian. *Int Food Res J.* (2013) 20:3101–6.

[86] Gill CO. Active packaging in practice: meat. In *Novel food packaging techniques.* Sawston: Woodhead Publishing Limited (2003). 10.1533/9781855737020.3.365

[87] Ratsimba A Rakoto D Jeannoda V Andriamampianina H Talon R Leroy S Physicochemical and microbiological characteristics of kitoza, a traditional salted/dried/smoked meat product of Madagascar. *Food Sci Nutri.* (2019) 7:2666–2273. 10.1002/fsn3.1122 31428353 PMC6694416

[88] Dzimba F Faria J de AF Walter EHM. Testing the sensory acceptability of biltong formulated with different spices. *Afr J Agricul Res.* (2007) 2:574–7.

[89] Naidoo K Lindsay D. Survival of Listeria monocytogenes, and enterotoxin-producing *Staphylococcus aureus* and *Staphylococcus pasteuri*, during two types of biltong-manufacturing processes. *Food Cont.* (2010) 21:1042–50. 10.1016/j.foodcont.2009.12.025

[90] Ajiboye A Kolawole O Oladosu T Adedayo M Akintunde J. Studies on the microorganisms associated with dried meat (Tinko) sold in Ilorin, Nigeria. *Afr J Microb Res.* (2011) 16:4150–54. 10.5897/ajmr11.419

[91] Menéndez RA Rendueles E Sanz JJ Santos JA García-Fernández MC. Physicochemical and microbiological characteristics of diverse Spanish cured meat products. *CYTA – J Food.* (2018) 16:199–204. 10.1080/19476337.2017.1379560

[92] Bader R Becila S Ruiz P Djeghim F Sanah I Boudjellal A Physicochemical and microbiological characteristics of El-Guedid from meat of different animal species. *Meat Sci.* (2021) 171:108277. 10.1016/j.meatsci.2020.108277 32805642

[93] Parra V Viguera J Sánchez J Peinado J Espárrago F Gutierrez JI Modified atmosphere packaging and vacuum packaging for long period chilled storage of dry-cured Iberian ham. *Meat Sci.* (2010) 84:760–8. 10.1016/j.meatsci.2009.11.013 20374854

[94] Seong PN Park KM Kang GH Cho SH Park BY Ba H The impact of ripening time on technological quality traits, chemical change and sensory characteristics of dry-cured loin. *Asian-Austr J Anim Sci.* (2015) 28:677–85. 10.5713/ajas.14.0789 25715685 PMC4412998

[95] Bellés M del Mar Campo M Roncalés P Beltrán JA. Supranutritional doses of vitamin E to improve lamb meat quality. *Meat Sci.* (2019) 149:14–23. 10.1016/j.meatsci.2018.11.002 30448473

[96] Hoffman JR Falvo MJ. Protein - Which is Best? *J Sports Sci Med.* (2004) 3:118–30. 24482589 PMC3905294

[97] Ahmad RS Imran A Hussain MB. Nutritional composition of meat. In: *Meat Science and Nutrition.* London: IntechOpen (2018). p. 61–77. 10.5772/intechopen.77045

[98] Kauffman R. Meat composition. In: Hui YH Nip WK Rogers R editors. *Meat science and applications.* Boca Raton, FL: CRC Press (2001). 10.1201/9780203908082.pt1

[99] Campbell RE Hunt MC Levis P Chambers IVE. Dry-aging effects on palatability of beef longissimus muscle. *J Food Sci.* (2001) 66:196–9. 10.1111/j.1365-2621.2001.tb11315.x

[100] Hwang YH Sabikun N Ismail I Joo ST. Changes in sensory compounds during dry aging of pork cuts. *Food Sci Anim Resour.* (2019) 39:379–87. 10.5851/kosfa.2019.e29 31304467 PMC6612786

[101] Li X Babol J Bredie WLP Nielsen B Tománková J Lundström K. A comparative study of beef quality after ageing longissimus muscle using a dry ageing bag, traditional dry ageing or vacuum package ageing. *Meat Sci.* (2014) 97:433–42. 10.1016/j.meatsci.2014.03.014 24769099

[102] Kim S Lee HJ Kim M Yoon JW Shin DJ Jo C. Storage stability of vacuum-packaged dry-aged beef during refrigeration at 4^°^C. *Food Sci Anim Resour.* (2019) 39:266–75. 10.5851/kosfa.2019.e21 31149668 PMC6533398

[103] Li J Li Z Wang N Raghavan GSV Pei Y Song C Novel sensing technologies during the food drying process. *Food Eng Rev.* (2020) 12:121–48. 10.1007/s12393-020-09215-2

[104] Guo L Li Y Ding S Wang B Zhu Y Pang B Effect of fermentation with two molds on characteristics of chicken meat. *J Food Qual.* (2021) 2021:1–9. 10.1155/2021/8845552

[105] Ingham SC Searls G Mohanan S Buege DR. Survival of *Staphylococcus aureus* and *Listeria monocytogenes* on vacuum-packaged beef jerky and related products stored at 21^°^C. *J Food Protec.* (2006) 69:2263–7. 10.4315/0362-028X-69.9.2263 16995535

[106] Park Y. Moisture and Water Activity. In: Leo ML Toldra NF editors. *Handbook of processed meats and poultry analysis.* Boca Raton, FL: CRC Press (2008). p. 35–67.

[107] Mauer LJ Bradley RL. Moisture and Total Solids Analysis. In: Nielsen SS editor. *Food analysis. food science text series.* Cham: Springer (2017). p. 257–86. 10.1007/978-3-319-45776-5_15

[108] Listrat A Gagaoua M Normand J Gruffat D Andueza D Mairesse G Contribution of connective to tissue components, muscle fibres and marbling to beef tenderness variability in longissimus thoracis, rectus abdominis, semimembranosus and semitendinosus muscles. *J Sci Food Agric.* (2020) 100:2502–11. 10.1002/jsfa.10275 31960978

[109] Kaur L Hui SX Morton JD Kaur R Chian FM Boland M. Endogenous proteolytic systems and meat tenderness: Influence of post-mortem storage and processing. *Food Sci Anim Resour.* (2021) 41:589–607. 10.5851/kosfa.2021.e27 34291209 PMC8277181

[110] Kim JH Lee HJ Shin DM Kim TK Kim YB Choi YS. The dry-aging and heating effects on protein characteristics of beef longissiumus dorsi. *Korean J Food Sci Anim Resour.* (2018) 38:1101–8. 10.5851/kosfa.2018.e43 30479515 PMC6238036

[111] Maqsood S Manheem K Gani A Abushelaibi A. Degradation of myofibrillar, sarcoplasmic and connective tissue proteins by plant proteolytic enzymes and their impact on camel meat tenderness. *J Food Sci Technol.* (2018) 55:3427–38. 10.1007/s13197-018-3251-6 30150801 PMC6098802

[112] Corral S Leitner E Siegmund B Flores M. Determination of sulfur and nitrogen compounds during the processing of dry fermented sausages and their relation to amino acid generation. *Food Chem.* (2016) 190:657–64. 10.1016/j.foodchem.2015.06.009 26213023

[113] Toldrá F. Characterization of Proteolysis. In *Dry-cured meat products. food nutri. press.* New York, NY: Wiley (2008). p. 113–34. 10.1002/9780470385111.ch6

[114] Toldrá F. *Dry-cured meat products.* New York, NY: Wiley (2002). p. 1–238. 10.1016/B978-0-08-100596-5.03014-6

[115] Bekhit A Morton JD Bhat ZF Kong L. Meat color: factors affecting color stability. *Encycloped Food Chem.* (2019) 2:202–10. 10.1016/B978-0-12-814026-0.21665-X

[116] Ferrier D Singh R Goyal R *Lippincott Illustrated Reviews: Biochemistry*. Philadelphia, PA: Lippincott Williams & Wilkins (2020).

[117] Wang X Wang Z Zhuang H Nasiru MM Yuan Y Zhang J Changes in color, myoglobin, and lipid oxidation in beef patties treated by dielectric barrier discharge cold plasma during storage. *Meat Sci.* (2021) 176:108456. 10.1016/j.meatsci.2021.108456 33621829

[118] Hoa VB Cho SH Seong PN Kang SM Kim YS Moon SS The significant influences of pH, temperature and fatty acids on meat myoglobin oxidation: a model study. *J Food Sci Technol.* (2020) 58:3972–80. 10.1007/s13197-020-04860-1 34471321 PMC8357901

[119] Gómez I Janardhanan R Ibañez FC Beriain MJ. The effects of processing and preservation technologies on meat quality: Sensory and nutritional aspects. *Foods.* (2020) 9:1416. 10.3390/foods9101416 33036478 PMC7601710

[120] Bulgaru V Popescu L Netreba N Ghendov-Mosanu A Sturza R. Assessment of quality indices and their influence on the texture profile in the dry-aging process of beef. *Foods.* (2022) 11:1526. 10.3390/foods11101526 35627098 PMC9141253

[121] Weston AR Rogers RW Althen TG. Review: The role of collagen in meat tenderness. *Prof Anim Sci.* (2002) 18:107–11. 10.15232/S1080-7446(15)31497-2

[122] Iida F Miyazaki Y Tsuyuki R Kato K Egusa A Ogoshi H Changes in taste compounds, breaking properties, and sensory attributes during dry aging of beef from Japanese black cattle. *Meat Sci.* (2016) 112:46–51. 10.1016/j.meatsci.2015.10.015 26519608

[123] Kim YHB Kemp R Samuelsson LM. Effects of dry-aging on meat quality attributes and metabolite profiles of beef loins. *Meat Sci.* (2016) 111:168–76. 10.1016/j.meatsci.2015.09.008 26437054

[124] Colle MJ Richard RP Killinger KM Bohlscheid JC Gray AR Loucks WI Influence of extended aging on beef quality characteristics and sensory perception of steaks from the gluteus medius and longissimus lumborum. *Meat Sci.* (2015) 110:32–9. 10.1016/j.meatsci.2015.06.013 26172241

[125] Kim JH Kim TK Shin DM Kim HW Kim YB Choi YS. Comparative effects of dry-aging and wet-aging on physicochemical properties and digestibility of Hanwoo beef. *Asian-Austr J Anim Sci.* (2020) 33:501–5. 10.5713/ajas.19.0031 31480178 PMC7054618

[126] Shahidi F Hossain A. Role of lipids in food flavor generation. *Molecules.* (2022) 27:5014. 10.3390/molecules27155014 35956962 PMC9370143

[127] Domínguez R Pateiro M Gagaoua M Barba FJ Zhang W Lorenzo JM. A comprehensive review on lipid oxidation in meat and meat products. *Antioxidants.* (2019) 8:429. 10.3390/antiox8100429 31557858 PMC6827023

[128] Cheng J. Lipid oxidation in meat. *J Nutri Food Sci.* (2016) 6:1–3. 10.4172/2155-9600.1000494

[129] Chaijan M Panpipat W. Mechanism of oxidation in foods of animal origin. In *Natural Antioxidants: Applications in Foods of Animal Origin.* Boca Raton, FL: Apple Academic Press (2017). p. 1–38. 10.1201/9781315365916-2

[130] Conte F Cincotta F Condurso C Verzera A Panebianco A. Odor emissions from raw meat of freshly slaughtered cattle during inspection. *Foods.* (2021) 10:2411. 10.3390/food10102411 PMC8535288 34681460

[131] Flores M Corral S Cano-García L Salvador A Belloch C. Yeast strains as potential aroma enhancers in dry fermented sausages. *Int J Food Microbiol.* (2015) 212:16–24. 10.1016/j.ijfoodmicro.2015.02.028 25765533

[132] Gómez M Lorenzo JM. Effect of fat level on physicochemical, volatile compounds and sensory characteristics of dry-ripened “chorizo” from Celta pig breed. *Meat Sci.* (2013) 95:658–66. 10.1016/j.meatsci.2013.06.005 23811106

[133] Lorenzo JM. Changes on physico-chemical, textural, lipolysis and volatile compounds during the manufacture of dry-cured foal “cecina”. *Meat Sci.* (2014) 96:256–63. 10.1016/j.meatsci.2013.06.026 23916960

[134] Perea-Sanz L Lopez-Diez JJ Belloch C Flores M. Counteracting the effect of reducing nitrate/nitrite levels on dry fermented sausage aroma by *Debaryomyces hansenii* inoculation. *Meat Sci.* (2020) 164:108103. 10.1016/j.meatsci.2020.108103 32145603

[135] Gou P Arnau J Garcia-Gil N Fulladosa ES. Dry−cured ham.. In *Handbook of meat and meat processing* Second ed. Boca Raton, FL: CRC Press (2012). p. 673–87. 10.1002/9780813820897.ch20

[136] Boateng EF Nasiru MM Agyemang M. Meat: Valuable animal-derived nutritional food. *A review.* Asian Food Sci J. (2020) 15:9–19. 10.9734/afsj/2020/v15i130140

[137] Mul JD Stanford KI Hirshman MF Goodyear LJ. Chapter two - exercise and regulation of carbohydrate metabolism. In *Molecular and cellular regulation of adaptation to exercise.* Cambridge, MA: Academic Press (2015). p. 17–37. 10.1016/bs.pmbts.2015.07.020 PMC4727532 26477909

[138] Daghlas SA Mohiuddin SS. Biochemistry, Glycogen. In *StatPearls.* Treasure Island, FL: StatPearls Publishing (2022). 30969624

[139] Chauhan SS England EM. Postmortem glycolysis and glycogenolysis: insights from species comparisons. *Meat Sci.* (2018) 144:118–26. 10.1016/j.meatsci.2018.06.021 29960720

[140] Keeton JT Ellerbeck SM Núñez, de González MT. Chemical composition. *Encycloped Meat Sci.* (2014) 1:235–43. 10.1016/B978-0-12-384731-7.00087-8

[141] Przybylski W Monin G Koćwin-Podsiadła M Krzêcio E. Glycogen metabolism in muscle and its effects on meat quality in pigs-A mini review. *Pol J Food Nutr Sci.* (2006) 15:257–62.

[142] Choe J. Overview of muscle metabolism, muscle fiber characteristics, and meat quality. *Korean J Agric Sci.* (2018) 45:50–7. 10.7744/kjoas.20180012

[143] Khan MI Jung S Nam KC Jo C. Postmortem aging of beef with a special reference to the dry aging. *Korean J Food Sci Anim Resour.* (2016) 36:159–69. 10.5851/kosfa.2016.36.2.159 27194923 PMC4869541

[144] Bhat ZF Morton JD Mason SL Bekhit AEA. Role of calpain system in meat tenderness: A review. *Food Sci Hum Wellness.* (2018) 7:196–204. 10.1016/j.fshw.2018.08.002

[145] Lofgren PA. Meat, poultry and meat products. In *Encyclopedia of Human Nutrition.* Oxford: Academic Press (2005). p. 230–7. 10.1016/B0-12-226694-3/00204-0

[146] Cilla A Alegría A Barberá R Guadalupe GL Toldrá F. Micronutrients and other minor meat components. In *Encyclopedia of Meat Sciences.* Cambridge, MA: Academic Press (2014). p. 212–6. 10.1016/B978-0-12-384731-7.00058-1

[147] Tamburrano A Tavazzi B Calla CAM Amorini AM Lazzarino G Vinceti S Biochemical and nutritional characteristics of buffalo meat and potential implications on human health for a personalized nutrition. *Ital J Food Saf.* (2019) 8:8137. 10.4081/ijfs.2019.8317 31632933 PMC6784592

[148] Sasiadek ZZ Marchewka J Horbanczuk JO Wierzbicka A Lipinska P Jozwik A Nutrients composition in fit snacks made from ostrich, beef and chicken dried meat. *Molecules.* (2018) 23:1267. 10.3390/molecules23061267 PMC6099787 29799493

[149] Shi S Zhao M Li Y Kong B Liu Q Sun F Effect of hot air gradient drying on quality and appearance of beef jerky. *LWT.* (2021) 150:111974. 10.1016/j.lwt.2021.111974

[150] Eidson M Swell CM Graves G Olson R. Beef jerky gastroenteritis outbreaks. *J Environ Health.* (2000) 62:9.

[151] Nummer BA Harrison JA Harrison MA Kendall P Sofos JN Andress EL. Effects of preparation methods on the microbiological safety of home-dried meat jerky. *J Food Protect.* (2004) 67:2337–41. 10.4315/0362-028X-67.10.2337 15508655

[152] Dada TA Ekwomadu TI Mwanza M. Multi mycotoxin determination in dried beef using liquid chromatography coupled with triple quadrupole mass spectrometry (LC-MS/MS). *Toxins.* (2020) 12:1–14. 10.3390/toxins12060357 32485980 PMC7354427

[153] Wagacha JM Muthomi JW. Mycotoxin problem in Africa: Current status, implications to food safety and health and possible management strategies. *Int J Food Microbiol.* (2008) 124:1–12. 10.1016/j.ijfoodmicro.2008.01.008 18258326

[154] Gashaw M. Review on mycotoxins in feeds: Implications to livestock and human health. *J Agricul Res.* (2016) 5:137–44.

[155] Adeyeye SAO Yildiz F. Fungal mycotoxins in foods: A review. *Cogent Food Agricul.* (2016) 2:1213127. 10.1080/23311932.2016.1213127

[156] Cawthorn DM Steinman HA Hoffman LC. A high incidence of species substitution and mislabelling detected in meat products sold in South Africa. *Food Control.* (2013) 32:440–9. 10.1016/j.foodcont.2013.01.008

[157] Campbell JA Gaydos NJ Egolf SR Watson S. Fate of *Escherichia coli* O157:H7, *Salmonella* spp., and Listeria monocytogenes During Curing and Drying of Beef Bresaola. *Meat Muscle Biol.* (2021) 5:1–8. 10.22175/mmb.11621

[158] Melo L Torres F Guimarães J Soutelino M Cruz A Cortez M Study of consumer perception about low-sodium foods and characteristics related to perception and purchase of low-sodium spreadable processed cheese. *J Sens Stud.* (2021) 37:12–30. 10.1111/joss.12732

