# Supplementary material for: A comprehensive review of drying meat products and the associated effects and changes
Source: Front Nutr. 2022 Nov 28;9:1057366. doi: 10.3389/fnut.2022.1057366 (PMC9742493; doi:10.3389/fnut.2022.1057366)
Supplement: Supplementary file 1 [file Table_1.docx]

**Supplementary Table 1.** Summarised for the main component in meat composition.

| **Component** | **Percentage / Quantity** |
| --- | --- |
| Water  Bound  Immobilised  Free | 75%  Largest proportion |
| Protein  Myofibrillar (Myosin)  Sarcoplasmic (Myoglobin)  Connective tissue (Collagen) | 19%  50-55%  30-34%  10-15% |
| Lipid  Triglycerides (high in SFA)  Phospholipids (high in PUFA) | 2.5%  >90%, more SFA  <10%, more PFA |
| Carbohydrate  Glycogen | 1.2%  Most predominant form in muscle |
| Nitrogen compound  Taurine  Alanine, Glutamic | 1.65%  0.02-0.1%  0.01-0.05% |
| Vitamins  Water-soluble :B1,B2,B3,B6,B12  Fat-soluble: A,D | Range: µg-mg  0.03–40 mg/100 g |
| Minerals  Iron  Zinc  Phosphorus  Potassium  Magnesium  Copper  Selenium  Calcium  Sodium | Mostly found in red meat  Mostly found in red meat |
