# Supplementary material for: A comprehensive review of drying meat products and the associated effects and changes
Source: Front Nutr. 2022 Nov 28;9:1057366. doi: 10.3389/fnut.2022.1057366 (PMC9742493; doi:10.3389/fnut.2022.1057366)
Supplement: Supplementary file 2 [file Table_2.docx]

**Supplementary Table 2.** Factors involved in the safety of dried meat products.

| **Factors involved in safeness of dried meat** | **Optimal conditions to ensure safeness of dried meat** | **References** |
| --- | --- | --- |
| Temperature | Higher temperatures help to kill unwanted microorganisms. | [57, 148] |
| Meat curing | Curing meats before drying them can make the meat less favourable as a nutrient source for microorganisms. |  |
| Method of drying | Drying methods in which the meat is exposed to the environment (sun drying) increases the risk of contamination in the dried meat products. |  |
| Adulteration | The presence of undeclared substances in dried meat products can pose as a threat to health, considering allergies. | [154] |
